# Deprivation of EGFR signal causes senolysis in PDAC with CDK4/6 inhibition

**DOI:** 10.1038/s41418-025-01634-0

**Published:** 2025-12-18

**Authors:** Yuanyuan Zhang, Susumu Kohno, Keqi Gao, Mahadi Hasan, Tomohisa Baba, Zixue Zhang, Nao Sankoda, Hai Yu, Junjian Pan, Noriko Gotoh, Makoto Nakanishi, Yasuhiro Yamada, Jindan Sheng, Takiko Daikoku, Yoshikazu Johmura, Chiaki Takahashi

**Affiliations:** 1 Division of Oncology and Molecular Biology, Cancer Research Institute, Kanazawa University, Kanazawa, Ishikawa Japan; 2 Division of Cancer and Senescence Biology, Cancer Research Institute, Kanazawa University, Kanazawa, Ishikawa Japan; 3 Division of Animal Disease Model, Research Center for Experimental Modeling of Human Disease, Kanazawa University, Kanazawa, Ishikawa Japan; 4 Department of Public Health, Daffodil International University, Dhaka, Bangladesh; 5 Department of Molecular Pathology, Graduate School of Medicine, The University of Tokyo, Bunkyo-ku, Tokyo Japan; 6 Division of Cancer Cell Biology, Cancer Research Institute, Kanazawa University, Kanazawa, Ishikawa Japan; 7 Division of Cancer Cell Biology, Institute of Medical Science, The University of Tokyo, Minato-ku, Tokyo Japan; 8 Maternal-Fetal Medicine and Gynecologic Oncology, Shanghai First Maternity and Infant Hospital, School of Medicine, Tongji University, Shanghai, China; 9 Department of Gynecology, Shanghai First Maternity and Infant Hospital, School of Medicine, Tongji University, Shanghai, China; 10 Dana-Farber Cancer Institute, Harvard Medical School, Boston, MA USA

**Keywords:** Preclinical research, Tumour-suppressor proteins

## Abstract

Approved KRAS inhibitors have shown limited therapeutic benefit over standard chemotherapy in PDAC and often encounter acquired resistance due to additional genetic alterations. RAS and RB1 functionally antagonize each other, which explains why RB1 is rarely mutated in KRAS-driven tumors. In PDAC cells, CDK4/6 inhibition induced cellular senescence accompanied by partial apoptosis. However, additional treatment with a senolytic agent or an ERK inhibitor promoted more efficient tumor cell elimination. While CDK4/6 inhibition downregulated KRAS activity, it concurrently upregulated EGFR signaling in a SASP and JNK-dependent manner. Deprivation of EGFR signaling after CDK4/6 inhibition triggered apoptosis in senescent cells in a manner similar to the treatment with a senolytic agent. In contrast, specific inhibition of KRAS induced modest enhancement of EGFR activity and SASP in a JNK-independent manner. Collectively, our study proposes that the CDK4/6 inhibitor may achieve greater therapeutic efficacy when combined with the EGFR inhibitor than KRAS inhibitor monotherapy.

## Introduction

The combination therapy by cyclin-dependent kinase (CDK) 4/6 inhibition and estrogen receptor (ER) pathway blockade has been highly successful in prolonging the progression-free survival compared with endocrine therapy alone in patients with advanced hormone receptor (HR)-positive, human epidermal growth factor receptor 2 (HER2)-negative breast cancer [1, 2]. ER pathway inhibition reduces D-type cyclin expression, thereby attenuating cyclin–CDK4/6 activity. Thus, the combination of a CDK4/6 inhibitor and endocrine therapy simultaneously targets two critical nodes within the same signaling pathway in HR-dependent breast cancer.

Except for retinoblastoma and small cell lung cancer (SCLC), the loss of retinoblastoma 1 (RB1) function typically occurs during tumor progression. Approximately 70% of malignant tumors retain an intact RB1 gene, providing a strong rationale for the therapeutic use of CDK4/6 inhibitors [3, 4]. Multiple studies have demonstrated that CDK4/6 inhibition, by maintaining RB1 in an unphosphorylated state, induces G1 arrest, spontaneous differentiation, enhanced immunogenicity, and cellular senescence but only limited apoptosis [5–9].

Oncogenic KRAS mutations strongly suppress RB1 function by upregulating cyclin D1 and subsequently activating the cyclin–CDK4/6 complex [10–12]. Inversely, RB1 has been reported to suppress RAS activity [13, 14]. Epistatic studies in *C. elegans* first suggested that the RB1 orthologue (*lin-35*) can regulate the activation status of the RAS orthologue (*let-23*) [15]. The hypothesis that RB1 could act upstream of RAS has been extensively investigated in biochemical studies [12] as well as in mouse embryogenesis and tumorigenesis [16–18]. More recently, RB1 was shown to inhibit RAS maturation by downregulating isoprenylation [19]. The mutual antagonism between RB1 and RAS may explain the rarity of RB1 mutations in RAS-mutated cancers. Based on this framework, we hypothesized that RB1 activation by CDK inhibitors might modulate RAS activity even in the presence of oncogenic mutations.

The KRAS^G12C^ mutation, which can be directly targeted by G12C-specific KRAS inhibitors, is found in only ~1% of pancreatic ductal adenocarcinoma (PDAC) patients [20, 21]. Clinical approval of inhibitors for other KRAS mutations or of non-covalent pan-KRAS inhibitors is still pending [22–24]. In clinical trials for non-small cell lung cancer (NSCLC), additional mutations in KRAS, NRAS, or mitogen-activated protein kinase (MAPK) pathways are frequently observed [25–27], underscoring the need for alternative therapeutic strategies for PDAC that do not rely solely on direct targeting of oncogenic drivers. Besides the transactivation of cyclin D1 promoter by RAS signaling, PDACs commonly harbor deletions or inactivating mutations of cyclin-dependent kinase inhibitor 2 A (CDKN2A), resulting in de-repression of cyclin–CDK4/6 complexes [20]. Consequently, PDAC cells are often highly dependent on cyclin D1–CDK4/6 activity. Nevertheless, CDK4/6 inhibitor monotherapy has shown limited efficacy in PDAC [28, 29].

In this study, we screened a chemical library to identify compounds that synergize with a constitutively active RB1 mutant an unphosphorylatable truncated form of RB1 (RB7LP) to target KRAS-driven PDAC. From this screen, we identified an extracellular signal-regulated kinase (ERK) inhibitor, consistent with findings from a previous study [29]. To elucidate the underlying mechanism and establish a clinically relevant strategy, we analyzed time and dose-dependent changes in cellular signaling following the treatment with palbociclib. We observed a rapid increase in epidermal growth factor receptor (EGFR) activity prior to ERK phosphorylation, which was dependent on the increased abundance of EGFR ligands, as well as a gradual downregulation of KRAS activity, potentially mediated by the post-translational modification. The increased EGFR phosphorylation contributed to the induction and maintenance of cellular senescence and the senescence-associated secretory phenotype (SASP). Notably, the inhibition of EGFR activity abolished these senescence phenotypes and promoted cell death when combined with CDK4/6 or CDK2/4/6 inhibition. We further compared CDK4/6 inhibitors to KRAS inhibitors in terms of their regulation of oncogenic signaling in PDAC. Our findings presented suggest that the combination of a CDK4/6 inhibitor and an anti-EGFR antibody may be more effective than KRAS inhibitor monotherapy.

## Results

### Senolytic agent enhances cell death in PDAC cells treated with CDK4/6 inhibitor

**Fig. 1:**
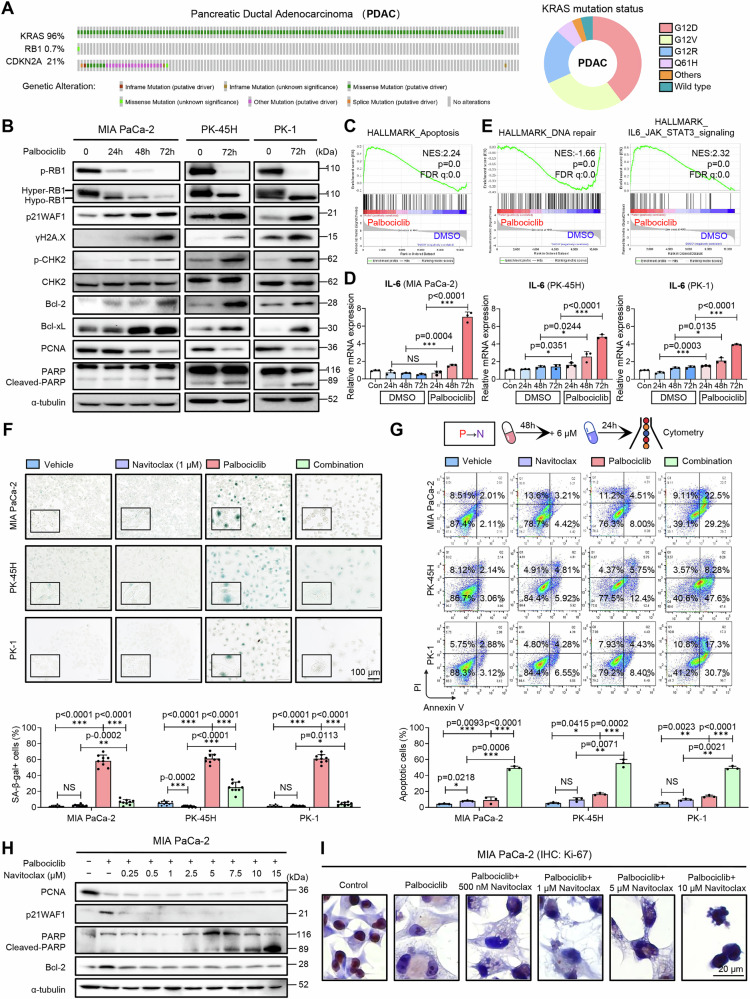
Senolytic agent enhances cell death in PDAC cells treated with CDK4/6 inhibitor. **A** Genetic alteration in PDAC patients according to publicly available transcriptome (The Cancer Genome Atlas [TCGA]) (left). Spectrum of KRAS mutations in human PDAC according to the cBioPortal database (https://www.cbioportal.org/) (right). **B** Immunoblotting (IB) of the indicated proteins in the indicated cells treated with or without 10 µM palbociclib for the indicated time. **C** Enrichment plot of HALLMARK_Apoptosis pathway from GSEA (MIA PaCa-2 cells). **D** RT-qPCR determination of IL-6 in the indicated cells treated with or without 10 µM palbociclib for 0-72 h. **E** Enrichment plots of HALLMARK_DNA repair and HALLMARK_IL6_JAK_STAT3_signaling pathway from GSEA (MIA PaCa-2 cells). **F** Representative images of SA-β-gal staining in the indicated cells pre-treated with or without 10 µM palbociclib for 48 h, thereafter with 1 µM navitoclax for 48 h. Scale bars, 100 µm. Quantitation of SA-β-gal positive cells from 3 or more randomly chosen fields. **G** Representative flow cytometry profiles of annexin V/PI double staining in the indicated cells pre-treated with or without 10 µM palbociclib for 48 h, thereafter with 6 µM navitoclax for 24 h. Quantitation of apoptotic cells (% = early apoptotic cells in Q2 + late apoptotic cells in Q3). **H** IB of the indicated proteins in MIA PaCa-2 cells pre-treated with or without 10 µM palbociclib for 48 h, thereafter with the indicated doses of navitoclax for 48 h. **I** Immunohistochemical (IHC) staining of Ki-67 in MIA PaCa-2 cells pre-treated with or without 10 µM palbociclib for 48 ,h thereafter with the indicated doses of navitoclax for 48 h. Scale bars, 20 µm. α-tubulin was used as a loading control. DMSO was used as vehicle. All data are presented as mean ± SD of three independent experiments. One-way ANOVA followed by Tukey’s post-hoc test was performed in (**D,** **F**, and **G**). *p < 0.05, **p < 0.01, ***p < 0.001.

KRAS^G12C^ mutation is rare in PDAC, thus currently most patients are not benefited by G12C-specific KRAS inhibitors (Fig. 1A). Additionally, clinical trials by G12C-specific KRAS inhibitors failed to show significant superiority over gemcitabine or FOLFIRINOX in KRAS^G12C^ PDAC [30–32]. RB1 mutation is extremely rare in PDAC (Fig. 1A). This could be at least partially due to the mutual functional suppression between RB1 and RAS [10–19]. We attempted to treat PDAC with CDK4/6 or CDK2/4/6 inhibitor. Treatment of three PDAC cell lines (MIA PaCa-2, PK-45H and PK-1) with a CDK4/6 inhibitor palbociclib (1 μM or 10 μM) resulted in suppression of colony formation with G1 arrest (Supplementary Fig. 1A and 1B). The immunoblotting (IB) analyses of three PDAC lines indicated that the effective dose of palbociclib (10 μM) induced complete elimination of phosphorylated form of RB1 but incomplete poly ADP-ribose polymerase (PARP) cleavage (Fig. 1B). Apoptotic pathways are activated in palbociclib-treated MIA PaCa-2 cells when analyzed by the gene set enrichment analysis (GSEA) of RNA-sequencing (RNA-seq) results (Fig. 1C). Consistently, the annexin V/PI staining detected apoptosis in less than 20% of population following single treatment in all three lines (Supplementary Fig. 1C). Quantitation of IL-6 mRNA and GSEA results (DNA repair and JAK-STAT pathway) predicted that cellular senescence was induced (Fig. 1D, E). Indeed, the senescence-associated β-galactosidase (SA-β-gal) assay confirmed that palbociclib induced cellular senescence in a time-dependent manner in all three PDAC cell lines (Supplementary Fig. 1D).

We observed the increase of cyclin-dependent kinase inhibitor 1 A (p21^WAF1^), phosphorylated H2A histone family member X (γH2A.X), phosphorylated checkpoint kinase 2 (CHK2), B-cell lymphoma-2 (Bcl-2), B-cell lymphoma-XL (Bcl-xL), and PARP cleavage similarly in all three PDAC lines after exposure to palbociclib (Fig. 1B). Gemcitabine is often used for inoperable or postoperative PDAC cases. Most probably due to the G1 enrichment induced by palbociclib, PDAC cells treated with palbociclib did not synergize with the additive treatment by gemcitabine suggesting that the combination of CDK4/6 inhibitor and chemotherapy yields no merit (Supplementary Fig. 2A). However, importantly, a Bcl-2 inhibitor navitoclax known as a senolytic drug [33, 34] strongly antagonized the cellular senescence induced by palbociclib at a lower dose (1 μM) (Fig. 1F) and significantly synergized with palbociclib in inducing cell death at higher doses (6~15 μM) in 3 lines of PDAC cells (Fig. 1G, H). The increasing dose (0.25 ~ 15 μM) of navitoclax in the presence of an effective dose of palbociclib (10 μM) induced cell death with suppression of proliferating cell nuclear antigen (PCNA) and without reversal of SA-β-gal activity (Fig. 1H, I). This also confirms that in the presence of palbociclib, 1 μM of navitoclax is insufficient to induce PARP cleavage, but 10 μM navitoclax is sufficient to induce PARP cleavage within 48 h (Fig. 1H). Importantly, p21^WAF1^ expression induced by palbociclib was downregulated even with 0.25 μM navitocla,x suggesting that the senescent state induced by palbociclib is readily perturbed by navitoclax (Fig. 1H). Navitoclax treatment at both 1 μM and 10 μM in the presence of 10 μM palbociclib exhibited time-dependent downregulation of PCNA expression (Supplementary Fig. 3A). The elevation of Bcl-2 level observed upon palbociclib treatment explains the increased sensitivity to navitoclax (Fig. 1B). These findings suggest that upon combinational treatment with palbociclib and navitoclax, PDAC cells in the senescent state directly undergo apoptosis without re-entering to cell cycle.

### CDK4/6 inhibitor sensitizes PDAC to ERK inhibitor

**Fig. 2:**
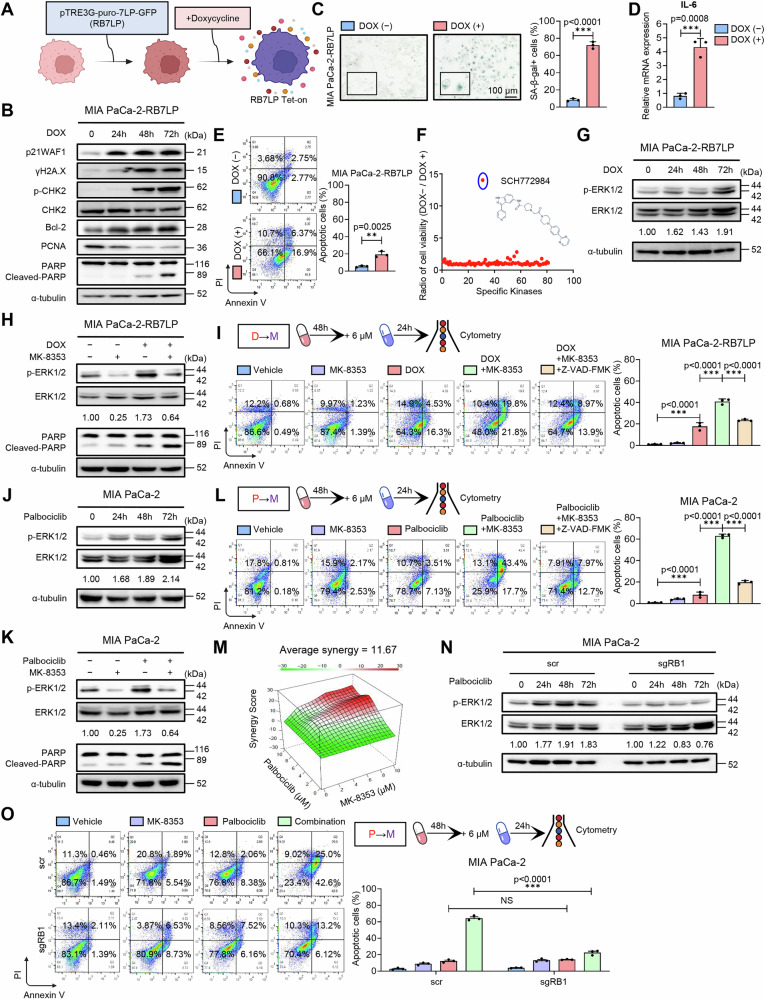
CDK4/6 inhibition sensitizes PDAC cells to ERK1/2 inhibitor. **A** Schematic diagram of Tet-induced expression of pTRE3G-puro-7LP-GFP (RB7LP). **B** IB of the indicated proteins in MIA PaCa-2-RB7LP treated with or without 1 μg/mL DOX for 0-72 h. **C** Representative images of SA-β-gal staining in MIA PaCa-2-RB7LP treated with or without 1 μg/mL DOX for 72 h. Scale bars, 100 µm. Quantitation of SA-β-gal positive cells from 3 or more randomly chosen fields. **D** RT-qPCR determination of IL-6 in MIA PaCa-2-RB7LP cells treated as in (**C**). **E** Representative flow cytometry profiles of annexin V/PI double staining in MIA PaCa-2-RB7LP cells treated as in (**C**). Quantitation of apoptotic cells (% = early apoptotic cells in Q2 + late apoptotic cells in Q3). **F** A screening of a chemical library in MIA PaCa-2-RB7LP cells. The compounds with higher selective toxicity against DOX(+) cells are detected with a higher index score. **G** IB of the indicated proteins in MIA PaCa-2-RB7LP cells treated as in (**B**). **H** IB of MIA PaCa-2-RB7LP cells pre-treated with or without 1 μg/mL DOX for 48 h thereafter with 6 µM MK-8353 for 24 h. **I** Representative flow cytometry profiles of annexin V/PI double staining in MIA PaCa-2-RB7LP cells pre-treated with or without 1 μg/mL DOX for 48 h thereafter with 6 μM MK-8353 and 10 μM Z-VAD-FMK for 24 h. Quantitation of apoptotic cells (% = early apoptotic cells in Q2 + late apoptotic cells in Q3). **J** IB of the indicated proteins in MIA PaCa-2 cells treated with or without 10 µM palbociclib for 0-72 h. **K** IB of MIA PaCa-2 cells pre-treated with or without 10 μM palbociclib for 48 h thereafter with 6 µM MK-8353 for 24 h. **L** Representative flow cytometry profiles of annexin V/PI double staining in MIA PaCa-2 cells pre-treated with or without 10 μM palbociclib for 48 h thereafter with 6 μM MK-8353 and 10 μM Z-VAD-FMK for 24 h. Quantitation of apoptotic cells (% = early apoptotic cells in Q2 + late apoptotic cells in Q3). **M** Combination index was assessed in MIA PaCa-2 cells treated with varied combinations of palbociclib and MK-8353. **N** IB of the indicated proteins in MIA PaCa-2 cells transduced with scramble (scr) or single guide RNA (sgRB1) treated with or without 10 µM palbociclib for 0-72 h. **O** Representative flow cytometry profiles of annexin V/PI double staining in MIA PaCa-2-scr cells and MIA PaCa-2-sgRB1 cells pre-treated with or without 10 μM palbociclib for 48 h, thereafter with 6 μM MK-8353 24 h. Quantitation of apoptotic cells (% = early apoptotic cells in Q2 + late apoptotic cells in Q3). α-tubulin was used as a loading control. DMSO was used as a vehicle. All data are presented as mean ± SD of three independent experiments. Unpaired two-tailed Student’s *t*-test was performed in (**C**, **D**, **E**). One-way ANOVA followed by Tukey’s post-hoc test was performed in (**I**, **L**, **O**). *p < 0.05, **p < 0.01, ***p < 0.001.

In our previous study, we demonstrated that overexpression of a constitutively active form mutant of RB1 (RB7LP) largely mimics the efficacy of palbociclib in RB1-intact hepatoblastoma and hepatocellular carcinoma cells [9]. Upon the induction of RB7LP in PDAC cells by the treatment with doxycycline (DOX) (Fig. 2A), MIA PaCa-2 cells started to exhibit senescent phenotypes, and G1 arrest exactly as seen in the cells treated with palbociclib (Supplementary Fig. 4A–D and Fig. 2B–D). In addition, exactly as seen in palbociclib, RB7LP induced partial cell death (Fig. 2B, E). We concluded that the RB7LP induction mimics the efficacy of palbociclib in PDAC cell,s as we previously observed in liver tumors [9].

To find compounds that enhance the efficacy of palbociclib in PDAC treatment, we screened a chemical library covering protein kinase inhibitors. After 48 h incubation in the absence or presence of 1 μg/mL DOX, MIA PaCa-2 cells carrying pTRE3G-puro-RB7LP-GFP were exposed to 80 kinase inhibitors individually at given concentrations for 24 h and assessed for cytotoxicity. An ERK inhibitor, SCH772984, was detected with a kill ratio 15 times higher in DOX (+) than DOX (-) (Fig. 2F). The RB7LP induction significantly elevated the ERK1/2 level and their phosphorylation in a time-dependent manner (Fig. 2G). In the following experiments, we used MK-8353, another ERK inhibitor that is more often used in clinical trials and is supposed to be more specific [35, 36]. MK-8353 synergized with RB7LP in killing MIA PaCa-2 cells, which was antagonized by Z-VAD-FMK a pan-caspase inhibitor (Fig. 2H, I). Treatment of MIA PaCa-2 cells by palbociclib also increased ERK level and activity in a time-dependent manner (Fig. 2J). We demonstrated that at a given concentration and time, ERK inhibition significantly enhanced the efficacy of the CDK4/6 inhibitor to induce cell death in MIA PaCa-2 cells (Fig. 2K, L). We finally addressed synergy between palbociclib and MK-8353 with a combination of various concentrations and concluded that these two agents synergistically kill PDAC cells (Fig. 2M). In addition, we observed that these two agents synergize in killing two other PDAC cell lines (PK-45H and PK-1) as well (Supplementary Fig. 4E). Therefore, we concluded that ERK inhibition synergizes with CDK4/6 inhibition in killing PDAC cells.

### RB1 is necessary for the response to combination therapy upon ERK inhibition

We then addressed the requirement of RB1 for the synergy between CDK4/6 and the ERK inhibitor. Deletion of RB1 in three KRAS PDAC lines significantly lessened the sensitivity to palbociclib in the colony formation assay (Supplementary Fig. 5A–D). Palbociclib treatment did not increase phosphorylated ERK in MIA PaCa-2 cells lacking RB1 (Fig. 2N). None of the three RB1-deleted PDAC lines allowed the exhibition of synergistic efficacy of CDK4/6 and ERK inhibitor (Fig. 2O and Supplementary Fig. 5E, F). Thus, the presence of RB1 is essential for PDAC cells to respond to the combination therapy.

### Suppression of mutant KRAS activity by CDK4/6 inhibitor

**Fig. 3:**
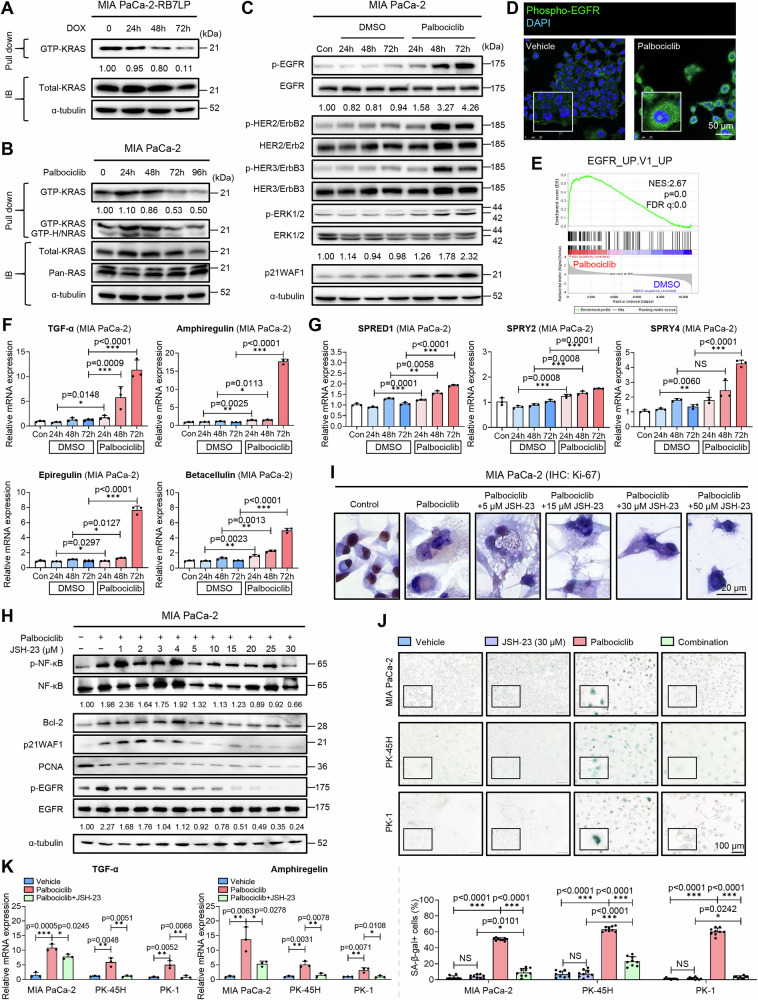
CDK4/6 inhibition induces SASP-mediated EGFR activation. **A**, **B** Pull down assay of GTP-KRAS in MIA PaCa-2-RB7LP cells or MIA PaCa-2 cells treated with or without 1 μg/mL DOX or 10 µM palbociclib for the indicated time. **C** IB of the indicated proteins in MIA PaCa-2 cells treated with or without 10 µM palbociclib for 0-72 h. **D** Representative immunofluorescence (IF) staining of phospho-EGFR in MIA PaCa-2 cells treated with or without 10 µM palbociclib for 72 h. Scale bars, 50 µm. **E** Enrichment plots of EGFR_UP.V1_UP pathway from GSEA (MIA PaCa-2 cells). **F**, **G** RT-qPCR determination of EGF family of ligands and Sprouty family members in MIA PaCa-2 cells treated as in (**C**). **H** IB of the indicated proteins in MIA PaCa-2 cells pre-treated with or without 10 µM palbociclib for 48 h thereafter with the indicated doses of JSH-23 for 48 h. **I** IHC of Ki-67 in MIA PaCa-2 cells pre-treated with or without 10 µM palbociclib for 48 h thereafter with the indicated doses of JSH-23 for 48 h. Scale bars, 20 µm. **J** Representative images of SA-β-gal staining in the indicated cells pre-treated with or without 10 µM palbociclib for 48 h, thereafter with 30 µM JSH-23 for 48 h. Scale bars, 100 µm. Quantitation of SA-β-gal positive cells from 3 or more randomly chosen fields. **K** RT-qPCR determination of TGF-α and Amphiregulin in the indicated cells pre-treated with or without 10 µM palbociclib for 48 h, thereafter with 30 µM JSH-23 for 48 h. α-tubulin was used as a loading control. DMSO was used as a vehicle. All data are presented as mean ± SD of three independent experiments. One-way ANOVA followed by Tukey’s post-hoc test was performed in (**F**, **G**, **J** and **K)**. *p < 0.05, **p < 0.01, ***p < 0.001.

We previously reported that the loss of RB1 function increases wild-type RAS activities by promoting protein isoprenylation, including farnesylation and geranylgenraylation, which are required for RAS protein maturation and activation [19]. Using a pull-down system with the RAS-binding domain of CRAF, we measured the abundance of GTP-loaded KRAS (Supplementary Fig. 6A, B). Induction of RB7LP in MIA PaCa-2 cells gradually decreased KRAS activity in 72 h (Fig. 3A). Palbociclib treatment as well, gradually decreased GTP-KRAS in 96 h (Fig. 3B). The pan-RAS antibody detected the transient, slight increase, then decrease in H/NRAS activity as well (Fig. 3B). These findings suggest that activation of RB1 by CDK4/6 inhibitor downregulates the amount of GTP-loaded KRAS^G12C^ as well as H/N-RAS in KRAS PDAC cells. The gradual downregulation of RAS activity is explainable by the time-dependent increase of unanchored RAS due to downregulated isoprenylation [19].

### CDK4/6 inhibitor induces SASP-mediated EGFR activation

To address why ERK activity is elevated by palbociclib despite mutated KRAS activity, we analyzed upstream of ERK. There are many signaling pathways known whereby ERK is activated independent of RAS status. For example, SRC or PLCγ can activate ERK in a RAS-independent manner [37, 38]. Given that, we next addressed the possible upstream of ERK. Palbociclib treatment induced elevated EGFR phosphorylation, followed by an increase of ERK activity in all of three PDAC lines (Fig. 3C and Supplementary Fig. 7A). HER2 and HER3 were activated (Fig. 3C). An immunofluorescent study confirmed the upregulation of EGFR phosphorylation following palbociclib treatment (Fig. 3D). The RNA-seq result was consistent with the activation of EGFR signaling (Fig. 3E). To address why EGFR activity is upregulated by palbociclib, we quantified the transcription of a number of genes possibly involved in EGFR activation. We discovered that many of the ligands for EGFR, especially those often overproduced upon the emergence of SASP, were upregulated in all three PDAC cell lines (Fig. 3F and Supplementary Fig. 7B). We observed slight upregulation of Sprouty family members, which unlikely explains EGFR signal activation (Fig. 3G).

We then examined the activity to stimulate EGFR in the conditioned media. We used DOX (−) and DOX (+) cells to avoid the effect of palbociclib carried over into the medium. The conditioned medium derived from DOX (+) induced EGFR activation significantly stronger than that from DOX (−) (Supplementary Fig. 7C). We noted that the conditioned medium derived from DOX (+) induced EGFR activation within 24 h. However, the transcriptional upregulation of EGFR ligands became apparent typically at 72 h. This timing is later than we observe upregulation of EGFR activity (24~48 h) following palbociclib treatment. Membrane-anchored EGFR ligands can be proteolytically released after various stimulation [39, 40]. This may well explain why EGFR activation occurs prior to transcriptional upregulation of EGFR ligands.

To investigate whether SASP underlies the mechanism of overproduction of EGFR ligands, we treated PDAC cells with palbociclib in combination with the increasing dose of JSH-23, an NF-κB inhibitor, which is supposed to antagonize SASP [41, 42]. Inhibition of NF-κB activity well correlated with downregulation of EGFR activity and p21^WAF1^ expression (Fig. 3H). SA-β-gal activity induced by palbociclib was antagonized by JSH-23 at a concentration sufficient to antagonize the EGFR activation in all three PDAC lines (Fig. 3I, J). In all three PDAC lines, JSH-23 antagonized overproduction of TGF-α and amphiregulin induced by palbociclib (Fig. 3K). All these findings indicate that NF-κB-mediated SASP induced by palbociclib causes overproduction of EGFR ligands.

### Deprivation of the EGFR signal synergizes with CDK4/6 inhibition

**Fig. 4:**
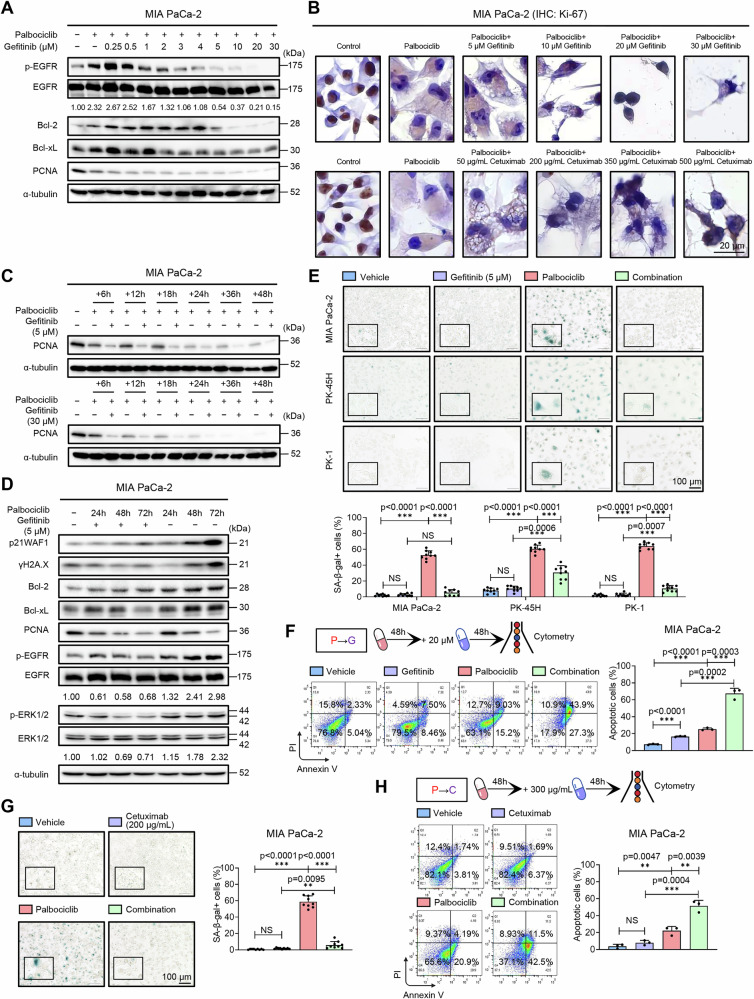
Deprivation of the EGFR signal synergizes with CDK4/6 inhibition. **A** IB of the indicated proteins in MIA PaCa-2 cells pre-treated with or without 10 µM palbociclib for 48 h, thereafter with the indicated doses of gefitinib for 48 h. **B** IHC of Ki-67 in MIA PaCa-2 cells pre-treated with or without 10 µM palbociclib for 48 h, thereafter with the indicated doses of gefitinib or cetuximab for 48 h. Scale bars, 20 µm. **C** IB of the indicated proteins in MIA PaCa-2 cells pre-treated with or without 10 µM palbociclib for 48 h, thereafter with the indicated doses of gefitinib for 0-48 h. **D** IB of the indicated proteins in the MIA PaCa-2 cells simultaneously treated with or without 10 µM palbociclib and 5 µM gefitinib for 0-72 h. **E** Representative images of SA-β-gal staining in the indicated cells pre-treated with or without 10 µM palbociclib for 48 h, thereafter with 5 µM gefitinib for 48 h. Scale bars, 100 µm. Quantitation of SA-β-gal positive cells from 3 or more randomly chosen fields. **F** Representative flow cytometry profiles of annexin V/PI double staining in MIA PaCa-2 cells pre-treated with or without 10 μM palbociclib for 48 h, thereafter with 20 µM gefitinib for 48 h (P → G). Quantitation of apoptotic cells (% = early apoptotic cells in Q2 + late apoptotic cells in Q3). **G** Representative images of SA-β-gal staining in MIA PaCa-2 cells pre-treated with or without 10 µM palbociclib for 48 h, thereafter with 200 µg/mL cetuximab for 48 h. Scale bars, 100 µm. Quantitation of SA-β-gal positive cells from 3 or more randomly chosen fields. **H** Representative flow cytometry profiles of annexin V/PI double staining in MIA PaCa-2 cells pre-treated with or without 10 μM palbociclib for 48 h, thereafter with 350 µg/mL cetuximab for 48 h. Quantitation of apoptotic cells (% = early apoptotic cells in Q2 + late apoptotic cells in Q3). α-tubulin was used as a loading control. DMSO was used as a vehicle. All data are shown as mean ± SD of three independent experiments. One-way ANOVA followed by Tukey’s post-hoc test was performed in **E**-**H**. *p < 0.05, **p < 0.01, ***p < 0.001.

Gefitinib can abolish the activity of EGFR even when it is wild type [43, 44]. We therefore treated PDAC cells with palbociclib in combination with the increasing dose of gefitinib. 5 μM gefitinib partially inhibited EGFR activity in MIA PaCa-2 cells, which was sufficient to suppress Ki-67 level, PCNA level, ERK activation, and SA-β-gal activity in the presence of palbociclib (Fig. 4A-E). 20 or 30 μM gefitinib, which almost completely abolishes EGFR activity, eliminated MIA PaCa-2 cells in the presence of palbociclib (Fig. 4A, B upper and F). At both 5 and 30 μM, gefitinib suppressed PCNA in the presence of palbociclib in a time-dependent manner (Fig. 4C). During the time course experiments, the increasing dose of gefitinib canceled cellular senescence and caused cells death without allowing cells to reenter cell cycle in a manner exactly same as navitoclax (Fig. 1H and I). Taken together, these findings indicate that EGFR signal deprivation causes cell death in a senolytic manner in PDAC cells senesced by CDK4/6 inhibition.

Importantly, cetuximab, a neutralizing antibody to EGFR, recapitulated the effect of gefitinib (Fig. 4B lower, G, H). These findings highlight the therapeutic benefit of a combination of CDK4/6 inhibitor and EGFR signal deprivation. As it is clinically hard to administer gefitinib to patients with EGFR-wild type tumors, cetuximab, which can efficiently target wild type EGFR, is a desirable option when designing therapy of PDAC patients with CDK4/6 inhibitor and EGFR inhibitor.

### The sequence of CDK4/6 inhibition and EGFR signal deprivation

A study of the combination of CDK4/6 inhibitor with chemotherapy in PDAC proposed that the CDK4/6 inhibitor should be administered after cytotoxic chemotherapy because it better prevents tumor cells recover from chromosomal damage [45]. In addition, the abovementioned data implicated that EGFR signal deprivation causes cell death in PDAC cells already senesced by CDK4/6 inhibition. We therefore addressed whether the sequence of administration influences the therapeutic efficacy in the case of our proposed combination therapy.

We found PDAC cells that have been removed of EGFR or related ligands (Amphiregulin and TGF-α) beforehand exhibit neither higher level of cellular senescence nor cell death when treated with palbociclib (Supplementary Fig. 8A-D and 9A). Similarly, the pretreatment of PDAC cells with gefitinib (G→P) prevented synergy between CDK4/6 inhibitor and gefitinib in colony formation suppression and cell death induction (Supplementary Fig. 9B-D). However, simultaneous (PG) or post-treatment by EGFR inhibitors following CDK4/6 inhibition (P→G) exhibited significant efficacy (Fig. 4F and Supplementary Fig. 9B-D). We confirmed that the simultaneous (PG) treatment exhibits significant synergy in the other two PDAC cell lines as well (Supplementary Fig. 9E). Although pretreatment by gefitinib (G→P) did not enhance the induction of cell death following the treatment by palbociclib (Supplementary Fig. 9D), this way of treatment did not induce a detectable level of senescence as well (Supplementary Fig. 9F-H), just as tested by EGFR pre-deletion (Supplementary Fig. 8A, C and D), providing further molecular mechanisms of synergy. These findings indicate that EGFR signal deprivation should not be done prior to the administration of the CDK4/6 inhibitor.

### RB1 is necessary for the response to combination therapy upon EGFR signal deprivation

We then addressed the requirement of RB1 for the synergistic action of CDK4/6 inhibition and EGFR signal deprivation. Deletion of RB1 in MIA PaCa-2 cells antagonized EGFR and ERK activation upon palbociclib treatment (Supplementary Fig. 10A and Fig. 2N). Neither palbociclib nor low dose gefitinib (5 μM) did not increase SA-β-gal activity in the RB1-deficient MIA PaCa-2 cells (Supplementary Fig. 10B). Moreover, the simultaneous treatment (PG) did not induce SA-β-gal activity in RB1-decifient MIA PaCa-2 cells (Supplementary Fig. 10B). Combination of regular dose palbociclib and high dose gefitinib (30 μM) failed to sufficiently kill MIA PaCa-2 cells lacking RB1 (Supplementary Fig. 10C). These findings suggest that RB1 is required for PDAC cells to respond to the combination therapy that we propose in this study.

We next addressed whether the newly developed CDK2/4/6 inhibitor PF06873600 (ebvaciclib) [46] exhibits any advantage over the CDK4/6 inhibitor palbociclib when combined with EGFR signal inhibitors. PF06873600 induced hypo-phosphorylation of RB1 slightly faster than palbociclib, however, elevation of ERK and EGFR activities took place at a similar level and timing (Supplementary Fig. 11A). In addition, induction of SASP factors and Sprouty family genes by PF06873600 was comparable to palbociclib (Supplementary Fig. 11B, C and Fig. 3F, G). Low dose gefitinib antagonized elevation of SA-β-gal activity upon treatment by PF06873600 similarly in the case of palbociclib in all tested PDAC lines (Supplementary Fig. 11D). These findings suggest that at least in the combination therapy of PDAC with EGFR signal inhibitors, CDK2/4/6 inhibitor may not have obvious advantage over CDK4/6 inhibitors and that we do not have to wait for the clinical development of CDK2/4/6 inhibitors at least for our proposed combination therapy.

### CDK4/6 and KRAS inhibitors commonly target many pathways to suppress PDAC

**Fig. 5:**
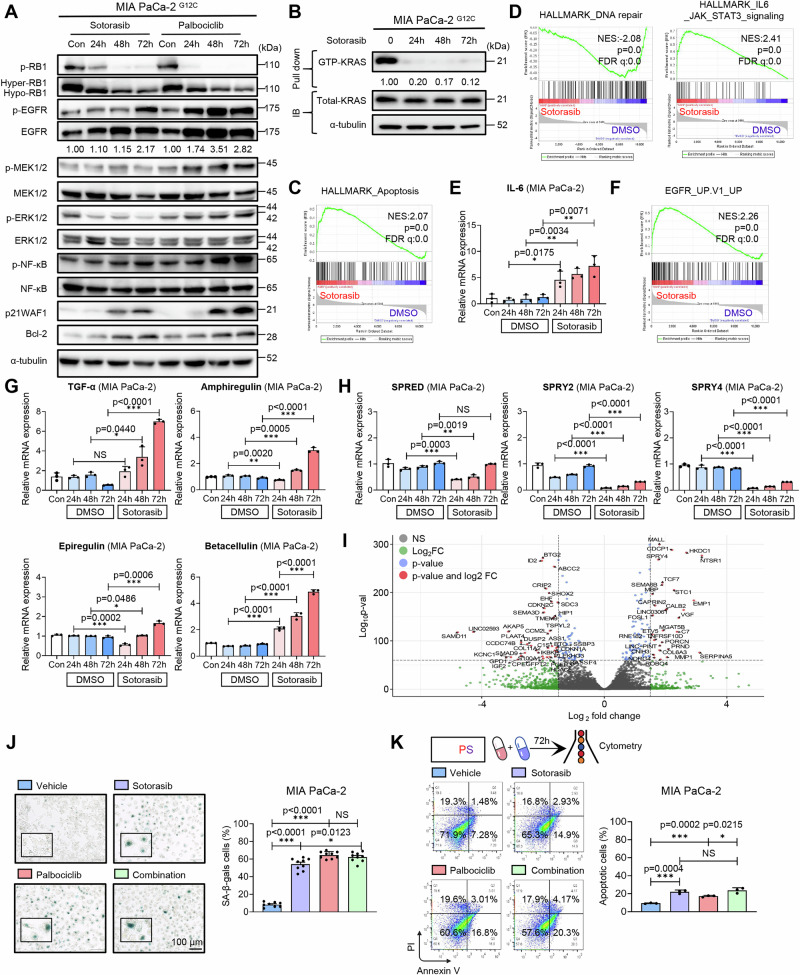
CDK4/6 and KRAS inhibitor commonly target many pathways to suppress PDAC. **A** IB of the indicated proteins in MIA PaCa-2 cells treated with or without 1 µM sotorasib or 10 µM palbociclib for 0-72 h. **B** Pull down assay of GTP-KRAS in MIA PaCa-2 cells treated with or without 1 µM sotorasib for 0-72 h. **C**, **D** Enrichment plots of HALLMARK_Apoptosis, HALLMARK_DNA repair, and HALLMARK_IL6_JAK_STAT3_signaling pathway from GSEA (MIA PaCa-2 cells). **E** RT-qPCR determination of IL-6 in MIA PaCa-2 cells treated with or without 1 µM sotorasib for 0-72 h. **F** Enrichment plots of EGFR_UP.V1_UP pathway from GSEA (MIA PaCa-2 cells). **G**, **H** RT-qPCR determination of EGF family of ligands and Sprouty family members in MIA PaCa-2 cells treated with or without 1 µM sotorasib for 0-72 h. **I** An enhanced volcano plot was created based on total fraction RNA-seq data [sotorasib (+) vs palbociclib (+)] using all differentially expressed genes (DEGs) (17,622 genes). Y-axis shows the mean expression value of log_10_ (*p*-value), and the x-axis displays the log_2_ fold change value. Red points: up-regulated DEGs; Blue points: down-regulated DEGs. **J** Representative images of SA-β-gal staining in MIA PaCa-2 cells simultaneously treated with or without 1 µM sotorasib and 10 μM palbociclib for 72 h. Quantitation of SA-β-gal positive cells from 3 or more randomly chosen fields. **K** Representative flow cytometry profiles of annexin V/PI double staining in MIA PaCa-2 cells treated as in (**J**). Quantitation of apoptotic cells (% = early apoptotic cells in Q2 + late apoptotic cells in Q3). α-tubulin was used as a loading control. DMSO was used as a vehicle. All data are presented as mean ± SD of three independent experiments. One-way ANOVA followed by Tukey’s post-hoc test was performed in (**G**, **H**, **J** and **K**). *p < 0.05, **p < 0.01, ***p < 0.001.

Now we attempt to figure out the superiority of CDK4/6 inhibitor-EGFR signal deprivation combination therapy over KRAS inhibitor monotherapy. In addition, RB1 status appeared to affect the activation status of oncogenically mutated KRAS in PDAC (Fig. 3A, B). We therefore needed to address signals commonly or differentially influenced by CDK4/6 and the KRAS inhibitor. To this end, we treated MIA PaCa-2 cells carrying KRAS^G12C^ mutation with sotorasib (AMG-510), a KRAS^G12C^ inhibitor. Sotorasib induced RB1 hypo-phosphorylation quickly, as seen in palbociclib. However, elevation of EGFR activity was less robust, and ERK activity was downregulated in contrast to palbociclib treatment (Fig. 5A).

As highly expected, the amount of GTP-loaded KRAS^G12C^ diminished immediately after the treatment with sotorasib (Fig. 5B). The colony formation was suppressed by sotorasib in a dose-dependent manner (Supplementary Fig. 12A). Apoptosis and DNA repair pathways were induced at a level comparable to palbociclib at the concentrations minimally required for 90% colony suppression (1 µM sotorasib and 10 µM palbociclib) (Figs. 5C, D and 1C, E; Supplementary Figs. 12A and 1A). IL-6 was induced by sotorasib significantly quicker than by palbociclib (Figs. 5E and 1D). When examined by GSEA, upregulation of EGFR signaling was similarly observed as when treated by palbociclib (Figs. 5F and 3E). In addition, similar to in the case of palbociclib, lack of RB1 abolished EGFR activation in response to sotorasib treatment, but did not antagonize downregulation of ERK activity by sotorasib (Supplementary Fig. 12B, C).

Importantly, the induction of EGFR ligands upon 1 µM sotorasib treatment was less robust than upon 10 µM palbociclib treatment (Figs. 5G and 3F; Supplementary Table 1). Consistent with ERK activity downregulation, Sprouty family members were all downregulated after sotorasib treatment (Fig. 5H). A volcano plot analysis of RNA-seq in 10 µM palbociclib and 1 µM sotorasib-treated PDAC cells disclosed that the number of SASP factors, including MMP-1 and VGF, is higher expressed in palbociclib-treated cells than in sotorasib-treated, cells which was consistent with the result of measurement of EGFR ligands by RT-qPCR (Fig. 5G, I). Indeed, although palbociclib and sotorasib induced the same frequency of SA-β-gal positive cells, the signal density seems to be higher in those treated with palbociclib (Fig. 5J). All these findings indicate that at the concentration attaining the same degree of colony suppression, palbociclib induces a higher level of cellular senescence than sotorasib, yet most of the mechanisms are common except the swiftness of KRAS downregulation and behavior of ERK.

Finally, we addressed whether palbociclib and sotorasib synergize in PDAC cells. We observed virtually no synergy between these two agents (Fig. 5K), suggesting that palbociclib and sotorasib largely share targets to suppress PDAC. Furthermore, gefitinib exhibited a synergy with sotorasib significantly less efficiently than with palbociclib (Supplementary Fig. 12D, E), suggesting that sotorasib-induced senescent phenotypes depend less on EGFR as compared to palbociclib-induced.

### JNK mediates CDK4/6 inhibitor but not KRAS inhibitor to induce cellular senescence

Next, we further attempted to clarify the difference in the nature of cellular senescence induced by palbociclib and sotorasib. Transcriptomic comparison of palbociclib-treated and sotorasib-treated PDAC cells at the concentrations both attaining 90% suppression of colony formation (1 µM sotorasib and 10 µM palbociclib) further revealed that the oxidative phosphorylation (OXPHOS)-related genes are significantly more upregulated in palbociclib-treated PDAC cells than in sotorasib-treated (Supplementary Fig. 13A). Consistently, we observed increased MitoSOX activity more frequently in palbociclib-treated cells (Supplementary Fig. 13B). The increase of MitoSOX activity was similarly suppressed by mitoquinone (MitoQ) in both palbociclib-treated and sotorasib-treated PDAC cells (Supplementary Fig. 13C). MitoQ significantly antagonized the increase in SA-β-gal activity induced either by sotorasib or palbociclib suggesting that OXPHOS mediates cellular senescence induced either by palbociclib or sotorasib (Supplementary Fig. 13G middle). Importantly, the c-Jun N-terminal kinase (JNK) was significantly more activated when treated by palbociclib than sotorasib (Supplementary Fig. 13D). However, AMPKα and p38 MAPK were similarly activated after treatment with either by sotorasib or palbociclib (Supplementary Fig. 13D).

We then addressed the significance of differential JNK activation following the treatment with palbociclib and sotorasib. Reportedly, a specific JNK inhibitor, SP600125, causes phenotypical changes of cellular senescence and triggers a rapid increase in mitochondrial reactive oxygen species [47]. MitoSOX activity elevation induced by sotorasib was not antagonized by SP600125, however, that was induced by palbociclib was significantly antagonized by SP600125 (Supplementary Fig. 13E, F). In the sotorasib-treated cells in which JNK activity has already been downregulated, additional treatment by SP600125 unaffected SA-β-gal level (Supplementary Fig. 13D, E, G lower). In a sharp contrast, SP600125 antagonized the elevation of JNK activity induced by palbociclib and significantly downregulated SA-β-gal activity (Supplementary Fig. 13D, E, G lower). These findings indicate that the CDK4/6 inhibitor, but not the KRAS inhibitor, depends on JNK to induce cellular senescence. The difference of the mechanism whereby cellular senescence is induced by palbociclib and sotorasib may explain the difference in the characteristics of SASP.

### Simultaneous inhibition of CDK4/6 and EGFR in vivo

**Fig. 6:**
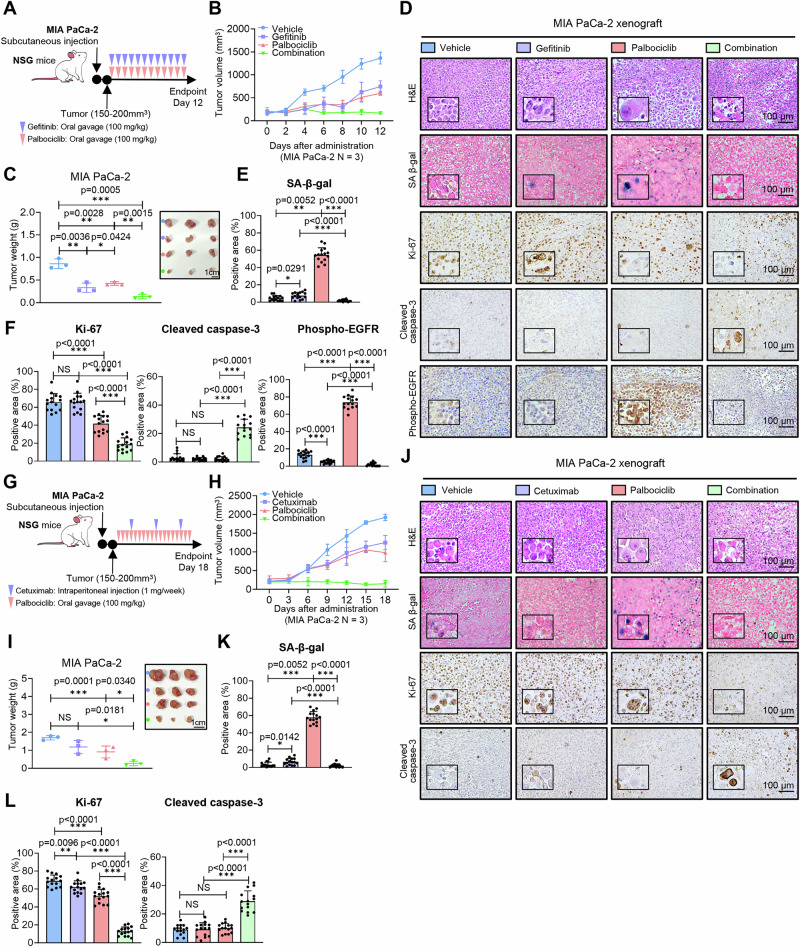
Simultaneuous inhibition of CDK4/6 and EGFR exhibits therapeutic efficacy in CDX model. **A** Schematic diagram of experiments using NSG mice subcutaneously xenografted with MIA PaCa-2 cells. Vehicle (corn oil or sodium L-lactate) or palbociclib (100 mg/kg, diluted in sodium L-lactate) together with gefitinib (100 mg/kg, diluted in corn oil) were given daily via oral gavage, and mice were euthanized at day 12 (*N* = 3). **B** Quantitation of volume of xenografts derived from NSG mice treated as in (**A**). Measurement of the tumor size was performed every 2 days. **C** Representative images and weights of xenografts at the endpoint. **D** SA-β-gal, H&E, and IHC staining of xenografts derived from NSG mice treated as in (**A**). Scale bars, 100 µm. **E**, **F** Quantitation of SA-β-gal staining and immunostaining of the indicated proteins of xenografts derived from NSG mice treated as in (**A**). 5 randomly chosen fields observed per mouse were quantified under 20×microscope. **G** Schematic diagram of experiments using NSG mice subcutaneously xenografted with MIA PaCa-2 cells. Vehicle (sodium L-lactate) or palbociclib (100 mg/kg, diluted in sodium L-lactate) was given daily via oral gavage. Cetuximab (1 mg/week) was given weekly by intraperitoneal injection (3 times in total). Mice were euthanized at day 18th (*N* = 3). **H** Quantitation of volume of xenografts derived from NSG mice treated as in (**G**). Measurement of the tumor size was performed every 2 days. **I** Representative images and weights of xenografts at the endpoint. **J** SA-β-gal, H&E, and IHC staining of xenografts derived from NSG mice treated as in (**G**). Scale bars, 100 µm. **K**, **L** Quantitation of SA-β-gal staining and immunostaining of the indicated proteins of xenografts derived from NSG mice treated as in (**G**). 5 randomly chosen fields observed per mouse were quantified under 20× microscope. All data are shown as mean ± SD of three independent experiments. One-way ANOVA followed by Tukey’s post-hoc test was performed in (**C**, **E**, **F**, **I**, **K** and **L**). *p < 0.05, **p < 0.01, ***p < 0.001.

Single usage of palbociclib (100 mg/Kg) or gefitinib (100 mg/Kg) allowed cell line (MIA PaCa-2)-derived xenograft (CDX) to continue to grow. However, the combined treatment significantly suppressed tumor growth (Fig. 6A-C). In mice, the *Cmax* of gefitinib when administered intravenously (10 mg/Kg) was reported to reach 4.40 ~ 4.96 μg/mL, which corresponds to 9.8 ~ 11.1 μM [48, 49]. The concentration of gefitinib that we typically used in in vitro assays was 20 μM. So, particularly in mice, we were supposed to attain the therapeutic concentration of gefitinib. The histological examination indicated that the SA-β-gal level was significantly upregulated in palbociclib-treated tumors; however, downregulated upon combination treatment (Fig. 6D, E). Moreover, the combined treatment significantly attenuated Ki-67 level and enhanced cleaved caspase-3 activation, which was not obvious in the single treatment (Fig. 6D, F). Phosphorylated EGFR was markedly upregulated in tumors developed in mice treated with palbociclib, which was significantly antagonized by additional treatment with gefitinib (Fig. 6D, F), confirming that the dose of palbociclib and gefitinib was at a sufficient therapeutic level in vivo. We examined another PDAC cell line, PK-1 for the assessment of the efficacy of combination therapy. The results recapitulated the results obtained by the examination of MIA PaCa-2 cells (Supplementary Fig. 14A–F). We then examined the combination of palbociclib and cetuximab, which exhibited a therapeutic efficacy completely comparable to the combination of palbociclib and gefitinib (Fig. 6G–L).

**Fig. 7:**
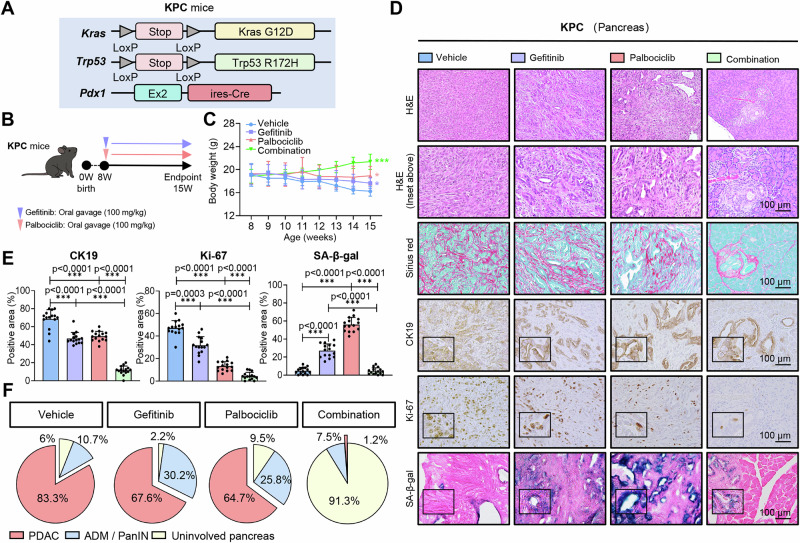
Simultaneuous inhibition of CDK4/6 and EGFR exhibits therapeutic efficacy in GEM model. **A** Design of KPC mice model used in this study. **B** Schematic of experiment using KPC mice. Treatment was initiated at the 8th week. Vehicle (corn oil or sodium L-lactate) or palbociclib (100 mg/kg, diluted in sodium L-lactate) and gefitinib (100 mg/kg, diluted in corn oil) was given via oral gavage 5 days per week. Mice were euthanized at 15th week post-birth (*N* = 5). **C** Quantitation of body weight of KPC mice treated as described in (**B**). **E** H&E, Sirius red, IHC, and SA-β-gal staining of pancreas in KPC mice treated as in (**B**). Scale bars, 100 µm. **E** Quantitation of immunostaining of the indicated proteins and SA-β-gal staining of 3 or more randomly chosen fields observed per mouse were quantified. **F** Quantitation of relative percentage of tissue phenotype (N = 5). The average values were recorded. All data are presented as mean ± SD. One-way ANOVA followed by Tukey’s post-hoc test was performed in (**C**, **E**). *p < 0.05, **p < 0.01, ***p < 0.001.

LSL-Kras^G12D^; LSL-Trp53^R172H^; Pdx1-ires-Cre (KPC) mice [50, 51] were treated with single or combined therapy for 3-8 weeks (Fig. 7A, B). Typically, by 8 to 10 weeks, KPC mice develop pancreatic intraepithelial neoplasia (PanIN) and focal PDAC. By 12 weeks, PDAC becomes more evident, and by 16 weeks, invasive PDAC associated with a dense desmoplastic reaction develops, which causes a decrease in body weight [50, 51]. PanIN is widely known to express a detectable level of SA-β-gal [52].

KPC mice continued to increase the body weight even during the combination therapy; however, monotherapies failed to rescue the loss of weight (Fig. 7C). Sirius red staining level collates with collagen fibrosis developed around PDAC and PanIN [53, 54]. Cytokeratin 19 (CK19) is a marker of primary and metastatic PDAC [55, 56]. Single treatments slightly attenuated fibrosis (Sirius red) and also slightly downregulated CK19 and Ki-67 expression (Fig. 7D, E). Palbociclib significantly upregulated SA-β-gal positive signal in PDAC lesion. Whereas the combination therapy strongly suppressed fibrosis (Sirius red), CK19, Ki-67, and SA-β-gal (Fig. 7D, E). The pathohistological examination indicated that the single usage of gefitinib or palbociclib significantly increased the PanIN region and slightly decreased the PDAC region (Fig. 7D, F). Gefitinib-treated tumors were SA-β-gal-negative in the PDAC region but as reportedly weakly SA-β-gal-positive in the PanIN region (Fig. 7D, E). However, the tumors treated with palbociclib were SA-β-gal-positive both in the PanIN and PDAC regions (Fig. 7D, E). The combination therapy significantly prevented both PanIN and PDAC development (Fig. 7D, F). The quantification of CK19 and Ki-67 staining indicated that the combination therapy is far superior to monotherapy (Fig. 7D–F).

Napsin A aspartic peptidase (NAPSA) is a marker specific to primary lung adenocarcinoma [57]. We observed a number of lung tumors in untreated KPC mice, which are CK19-positive and NAPSA-negative, indicating that those are metastasized from the primary PDAC (Supplementary Fig. 15A). The combination therapy significantly suppressed the development of lung tumors (Supplementary Fig. 15A–C). This finding indicates that the combination therapy proposed in this work is applicable to metastatic PDAC as well.

To examine whether the therapeutic dose of palbociclib induces cellular senescence in normal tissues, p16-Cre^ERT2^-tdTomato mice [58] were treated either by vehicle or palbociclib (125 mg/kg body weight) for 28 days and two intraperitoneal injections of tamoxifen (80 mg/kg body weight) (Supplementary Fig. 16A, B). After the treatment, tdTomato expression was traced. The body weight of mice in the palbociclib-treated group exhibited no statistically significant difference compared to the vehicle-treated group (Supplementary Fig. 16C). We could not discern treated from untreated in the number of tissues with respect to p16^Ink4a^ induction, despite the suppression of erythropoiesis and leukopoiesis being apparent (Supplementary Fig. 16D), indicating that therapeutic dose palbociclib does not induce detectable level cellular senescence in normal tissues but in PDAC (Supplementary Fig. 16E, F). These findings indicate that the CDK4/6 inhibitor treatment induces cellular senescence specifically in tumor cells.

## Discussion

The treatment with KRAS inhibitors is often hindered by the intrinsic resistance associated with KEAP1 mutations [59] or by acquired resistance caused by secondary oncogenic mutations in various RAS pathway genes [25–27].　When KRAS inhibitor therapy fails, it is crucial to establish alternative treatment strategies. CDK4/6 and CDK2/4/6 inhibitors activate RB1 when it remains intact. In HR-positive breast cancer, the combination of a CDK4/6 inhibitor with either an aromatase inhibitor or an estrogen antagonist is currently the first-line therapy [60, 61]. In other cancer types, combination treatments involving CDK4/6 inhibitors are primarily explored at the preclinical stage. Among these, NSCLC has garnered significant attention, with various combination strategies under investigation [62, 63]. Oncogenic RAS mutations rarely co-exist with RB1 mutations and strongly suppress RB1 function through hyperphosphorylation achieved in a D-type cyclin-dependent manner [10–19]. We here propose the clinical application of our combination therapy strategy for KRAS-driven cancers.

A direct comparison of KRAS^G12C^ inhibitors and CDK4/6 inhibitors in MIA PaCa-2 cells, which harbor the G12C mutation, provided valuable insights. Treatment with either inhibitor alone induced comparable levels of SA-β-gal activity in these cells. However, CDK4/6 inhibition led to a greater upregulation of OXPHOS and SASP, likely due to similar kinetics of RB1 activation but distinct kinetics of KRAS inactivation and JNK activation. In sharp contrast to CDK4/6 inhibition, which markedly upregulated JNK, KRAS inhibition downregulated it, highlighting the distinct signaling targets of these agents.　 We did not examine the combination of these treatments with immune checkpoint inhibitors in this study. However, based on the presented rationale, we propose a combinatorial therapeutic strategy using a clinically approved CDK4/6 inhibitor in conjunction with an EGFR inhibitor, such as cetuximab, for PDAC patients in whom KRAS inhibitor therapy becomes problematic.　 Furthermore, suppression of KRAS activity by CDK4/6 inhibition, through a mechanism distinct from the direct KRAS inhibition, may lessen the likelihood of additional mutations emerging in RAS genes.

## Materials and Methods

### Cell culture

MIA PaCa-2, PK-45H, PK-1, and HEK293T cells (RRID: CVCL_0428, CVCL_6748, CVCL_4717, CVCL_0063, RIKEN BRC Cell Bank) were cultured in D-MEM (043-30085, Wako) or RPMI-1640 (187-02021, Wako) containing 10% fetal bovine serum (AXB30120, Thermo Fisher Scientific) and 1% penicillin-streptomycin (16823291, Wako). All the cell lines confirmed negative for mycoplasma.

### Immunoblotting

Protein extraction and immunoblotting were performed as previously described [9]. Primary and secondary antibodies used are listed in Supplementary Table 2. Blots were visualized using FUSION-FX (Vilber Lourmat).

### Colony formation assay

Cells were seeded at a density of 2,500 cells per well in 60 mm culture dishes. After treatment with the indicated agents, cells were fixed in 4% paraformaldehyde (PFA) for 30 min at room temperature and stained with 0.1% crystal violet solution for 15 min, then thoroughly washed with distilled water. Plates were air-dried prior to imaging. Colony quantification was imaged using GT-X900 (EPSON) and performed using ImageJ (RRID:SCR_003070) by counting visible stained colonies.

### Flow cytometric analysis of cell cycle, apoptosis, and mitochondrial ROS

Resuspended cells were treated using FxCycle™ PI/RNase Staining Solution (F10797, Thermo Fisher Scientific), eBioscience Annexin V-FITC Apoptosis Detection Kit (BMS500FI-300, Thermo Fisher Scientific) and MitoSOX™ Red Mitochondrial Superoxide Indicator (M36008, Thermo Fisher Scientific), according to methods provided by manufacturer and processed on Flow cytometer Canto II (BD Biosciences, RRID: SCR_018056) and analyzed using FlowJO (RRID: SCR_008520).

### Reverse Transcription-Quantitative PCR

Total RNA from cells was extracted using TRIzol™ Reagent (15596018, Thermo Fisher Scientific) and reverse-transcribed into cDNA with the PrimeScript RT Reagent Kit (PR037A, Takara). RT-qPCR was performed on a LightCycler480 (Roche, RRID: SCR_018626). The Taqman probes and primers used in this study are listed in Supplementary Table 3, 4. The relative mRNA expression levels were normalized to the levels of human β actin [9].

### SA-β-gal assay

SA-β-gal staining of cultured cells was performed as previously described [9]. The dissected pancreas of genetically engineered mice and xenografted tumors were collected and fixed in 4% PFA. Tissues were sequentially immersed in 10%, 20% then 30% sucrose (30404-45, Nacalai Tesque)-PBS solutions until the tissue sank. Dehydrated tissues were embedded in Tissue-Tek O.C.T compound (4583, Sakura) and immersed in liquid nitrogen, then serially sectioned into 10 μm-thick sections and stained according to the procedure previously described [19]. Counterstaining was performed using eosin (9135-4P, Sakura). SA-β-gal positive cells in a 20×microscope were counted and averaged.

### Immunohistochemistry

The cells seeded on the chamber slides (177372, Thermo Fisher Scientific) were fixed with 4% PFA, pre-treated with 2.5% normal horse serum (MP-7401, VectorLabs, RRID: AB_2336529) and then incubated overnight with anti-Ki-67 antibody (9027, Cell Signaling Technology, RRID: AB_2636984) diluted at 1:400 in Can Get Signal® immunostain Immunoreaction Enhancer Solution (NKB-601, TOYOBO). Subsequently, the cells were incubated with anti-rabbit secondary antibody (MP-7401, VectorLabs, RRID: AB_2336529). Slides were stained with DAB (SK-4105, VectorLabs) and counterstained with hematoxylin (9135-4 P, Sakura). 5 μm-thick sections prepared from 4% PFA-fixed paraffin-embedded blocks were deparaffinized, hydrated, and underwent heat-induced antigen retrieval as previously described [9], followed by incubation with primary antibodies, thereafter with secondary antibodies listed in Supplementary Table 2 according to methods provided by the manufacturer. Subsequently, slides were stained with DAB (SK-4105, VectorLabs) and counterstained with hematoxylin (9135-4 P, Sakura).

### Tet-inducible RB7LP

The pTRE3G-puro-RB7LP-GFP vector was described previously [9].

### Cytotoxicity assay

Cell viability was assessed using a 0.5% trypan blue solution (29853-34, Nacalai Tesque) with a Bio-Rad TC20 Automated Cell Counter (RRID: SCR_025462).

### Chemical screening

We employed the IntelliScreen Highly Selective Kinase Inhibitor Library (10-7001, Focus Biomolecules). Inhibitors were dissolved in DMSO at 10 μM. Tested cells were placed onto 96-well plates (20,000 cells/well) containing compounds, making the final concentration at 1 μM. After 48 h incubation in the absence or presence of 1 μg/mL DOX, cells were exposed to the 80 kinase inhibitors individually at given concentrations for 24 h. The cell viability was assessed using Cell Counting Kit SF (07553-44, Nacalai Tesque) on an Infinite F200 Pro plate reader (Tecan). The sensitivity index of DOX (+) cells was determined by calculating the ratio of value A/value B, where value A was the viability of DOX (−) and value B was that of DOX (+) cells.

### Ras activity assay

The glutathione-S-transferases (GST)-fused RAS-binding domain (RBD) of CRAF was prepared as described previously [19]. Pull-down of GTP-KRAS using GST-CRAF-RBD proteins immobilized on glutathione-sepharose 4B (17-0756-01, GE HealthCare). Bound proteins and cell lysates were analyzed by SDS-PAGE and following IB with anti-KRAS antibody (33197, Cell Signaling Technology).

### Immunofluorescence

The cells seeded on the chamber slides (177372, Thermo Fisher Scientific) were fixed with 4% PFA, were pre-treated with blocking buffer (1X PBS/5% normal serum/0.3% Triton™ X-100) and then incubated overnight with phospho-EGFR antibody (3777, Cell Signaling Technology, RRID: AB_2096270) diluted at 1:500 in Can Get Signal® immunostain Immunoreaction Enhancer Solution (NKB-601, TOYOBO). Subsequently, the cells were incubated with anti-Rabbit IgG Alexa Fluor ^488^ (A-11034, Thermo Fisher Scientific, RRID: AB_2576217). DAPI (H-1200, VectorLabs) was used for counterstaining. Images were captured using SP8 LIGHTNING confocal microscope (Leica, RRID: SCR_018169). As for paraffin-embedded specimens, bone samples were pre-decalcified using the I.E.D. Unit (Biocare Medical). Sections were deparaffinized, hydrated, and underwent heat-induced antigen retrieval, followed by incubation with primary antibody anti-RFP (PM005, MBL, RRID: AB_591279) at 4**°**C overnight. All tissues except the intestine samples were washed and incubated with HRP-conjugated horse anti-rabbit secondary antibody (MP-7401, VectorLabs, RRID: AB_2336529). Fluorescent signals were amplified using the TSA Plus Fluorescein (Akoya Biosciences). Intestine samples were stained with anti-Rabbit IgG Alexa Fluor ^647^ (A-31573, Thermo Fisher Scientific, RRID: AB_2536183) after incubation with the primary antibody. Tissue slides were mounted using DAPI (H-1200, VectorLabs). Images were captured using a BZ X-710 fluorescent microscope (KEYENCE, RRID: SCR_017202).

### CRISPR/Cas9 system

We determined the target sequence for CRISPR-Cas9-mediated genome editing by using https://portals.broadinstitute.org/gpp/public/website. The oligonucleotides both forward and reverse, including the 20 bp target sequence and a BsmbI sticky end, were annealed and ligated into the pLentiCRISPRv2 (RRID: Addgene_48138). GRISPR/Cas9 sgRNA primers used in this study are listed in Supplementary Table 5.

### Animal studies

All procedures were performed in conformance with institutional guidelines and approved by the Institute for Experimental Animals and Use Committee of Kanazawa University. Six-week-old female NOD.Cg-PrkdcscidIl2rgtm1Wjl/SzJ (NSG) (The Jackson Laboratory, RRID: BCBC_4142) were inoculated with 5 × 10^6 target cells suspended in a 1:1 mixture of culture medium and Matrigel (Corning) (total volume: 100 μL) via subcutaneous injection into the right shoulder region. Drug administration was initiated when tumor volume exceeded 100 mm³. Body weight and tumor dimensions were measured three times weekly, with tumor volume calculated using the ellipsoid formula (length × width²)/2. LSL-Kras^G12D^; LSL-Trp53^R172H^; Pdx1-ires-Cre (KPC) mice were generated and genotyped as described previously [51]. Both male and female KPC mice were randomly assigned. Drug administration was initiated in 8-week-old mice. p16^Ink4a^-Cre^ERT2^-tdTomato mice were generated through homologous recombination in C57BL/6 embryonic stem cells with a targeting vector in which a firefly luciferase gene cassette is replaced by a Cre^ERT2^ gene cassette [58]. 18-week-old p16^Ink4a^-Cre^ERT2^-tdTomato female mice were randomly assigned. Mice were sacrificed after two intraperitoneal injections of tamoxifen (80 mg/kg). RBCs and WBCs counts were determined using an automated cell counter (MEK-6558, Nihon Kohden). Genotyping primers used in this study are shown in Supplementary Table 6.

### Chemical compounds

Chemical compounds used in this study are listed in Supplementary Table 7.

### Synergy analysis

The combination effect of two compounds at various concentration ranges was quantified with SynergyFinder supplied as an R package (R project, RRID: SCR_001905)(https://bioconductor.org/packages/release/bioc/html/synergyfinder.html). Synergy score based on the zeropotency interaction model is visualized as a three-dimensional landscape and indicated as a delta score.

### Statistical analysis

Data were analyzed by GraphPad Prism (RRID: SCR_002798) built-in tests and presented relative to their respective controls. Samples were selected randomly within each group. All the data were expressed as mean ± SD. Statistical significance between two groups was determined by an unpaired two-tailed Student’s *t*-test. Multiple groups were compared with one-way ANOVA followed by Tukey’s post-hoc test. *p* values < 0.05 were considered significant.

## Supplementary information

Supplementary FIGURES

Supplementary TABLES

Supplementary data UNCROPPED IMMUNOBLOTTING

Supplementary information ABBREVIATIONS

## Data Availability

The raw data of RNA-sequencing used for GSEA analysis and volcano plot is available in the DNA Data Bank of Japan (PRJDB18381). All genetic materials used for this paper are available from the authors on request.
