# Supplementary material for: Deprivation of EGFR signal causes senolysis in PDAC with CDK4/6 inhibition
Source: Cell Death Differ. 2025 Dec 18;33(6):1218–33. doi: 10.1038/s41418-025-01634-0 (PMC13246951; doi:10.1038/s41418-025-01634-0)
Supplement: Supplementary file 1 — Supplementary FIGURES [file 41418_2025_1634_MOESM1_ESM.pdf]

SUPPLEMENTARY FIGURE

Deprivation of EGFR signal causes senolysis in PDAC with CDK4/6 inhibition

Yuanyuan Zhang[1], Susumu Kohno[1], Keqi Gao[2], Mahadi Hasan[3], Tomohisa Baba[2], Zixue Zhang[1,2], Nao Sankoda[4], Hai Yu [1], Junjian Pan [1], Noriko Gotoh[5], Makoto Nakanishi[6], Yasuhiro Yamada[4], Jindan Sheng[1,7,8,9], Takiko Daikoku[3], Yoshikazu Johmura[2] and Chiaki Takahashi[1,*]

[1]Division of Oncology and Molecular Biology, Cancer Research Institute, Kanazawa University, Kanazawa, Ishikawa 920-1192, Japan. [2]Division of Cancer and Senescence Biology, Cancer Research Institute, Kanazawa University, Kanazawa, Ishikawa 920-1192, Japan. [3]Division of Animal Disease Model, Research Center for Experimental Modeling of Human Disease, Kanazawa University, Kanazawa, Ishikawa 920-8640, Japan.[4]Department of Molecular Pathology, Graduate School of Medicine, The University of Tokyo, Bunkyo-ku, Tokyo 113-0033, Japan[5]Division of Cancer Cell Biology, Cancer Research Institute, Kanazawa University, Kanazawa, Ishikawa 920-1192, Japan.[6]Division of Cancer Cell Biology, Institute of Medical Science, The University of Tokyo, Tokyo, Minato-ku, 108-8639, Japan.[7]Maternal-Fetal Medicine and Gynecologic Oncology, Shanghai First Maternity and Infant Hospital, School of Medicine, Tongji University, Shanghai, 200092, China.[8]Department of Gynecology, Shanghai First Maternity and Infant Hospital, School of Medicine, Tongji University, Shanghai, 200092, China.

[9]Dana-Farber Cancer Institute, Harvard Medical School, Boston, MA 02215, USA.

* Corresponding author:

Chiaki Takahashi, Cancer Research Institute, Kanazawa University, Japan; Tel: +81-76-264-6750; Fax: +81-76-234-4521; E-mail: chtakaha@staff.kanazawa-u.ac.jp

SUPPLEMENTARY FIGURES

Zhang et al., Supplementary Figure 1

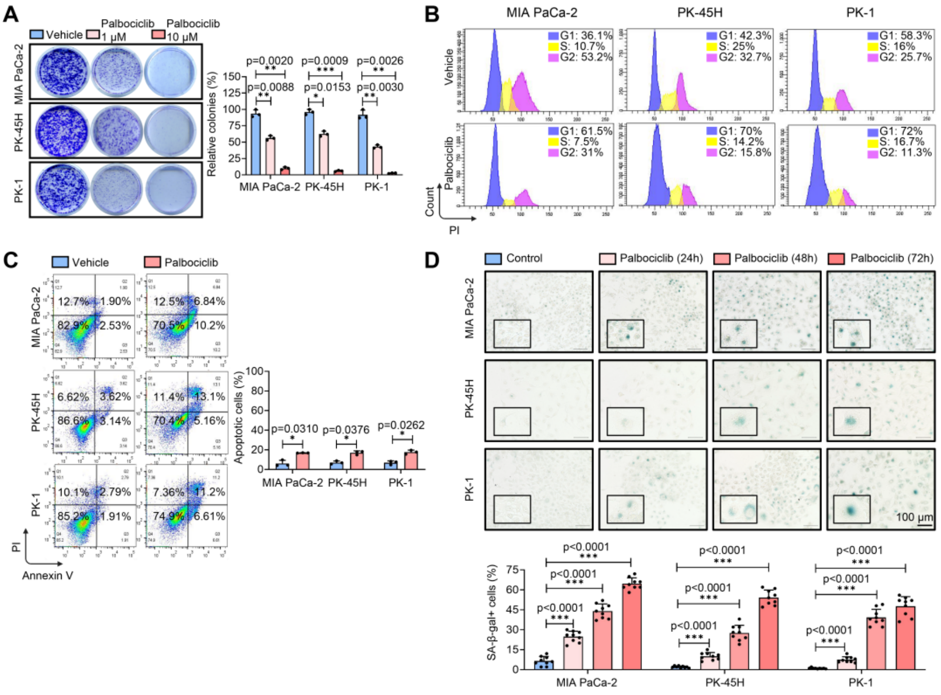

Supplementary Fig. 1 Senolytic agent enhances cell death in PDAC cells treated with CDK4/6 inhibitor. A Crystal violet staining of colonies from the indicated cell lines treated with or without the indicated doses of palbociclib. The relative colony number is normalized to vehicle (DMSO). **B** Representative flow cytometry profiles of FxCycle™ PI/RNase staining in the indicated cells treated with or without 10 μM palbociclib for 72h. **C** Representative flow cytometry profiles of annexin V/PI double staining in the indicated cells treated as in (**B**). Quantitation of apoptotic cells (% = early apoptotic cells in Q2 + late apoptotic cells in Q3). **D** Representative images of SA-β-gal staining in the indicated cells treated with or without 10 μM palbociclib for 0-72h. Scale bars, 100 μm. Quantitation of SA-β-gal positive cells from 3 or more randomly chosen fields. DMSO was used as vehicle. All

data are presented as mean ± SD of three independent experiments. One-way ANOVA followed by Tukey's post-hoc test was performed in **A**, **D**. Unpaired two-tailed Student's *t* test was performed in **C**. *$p < 0.05$, **$p < 0.01$, ***$p < 0.001$.

Zhang et al., Supplementary Figure 2

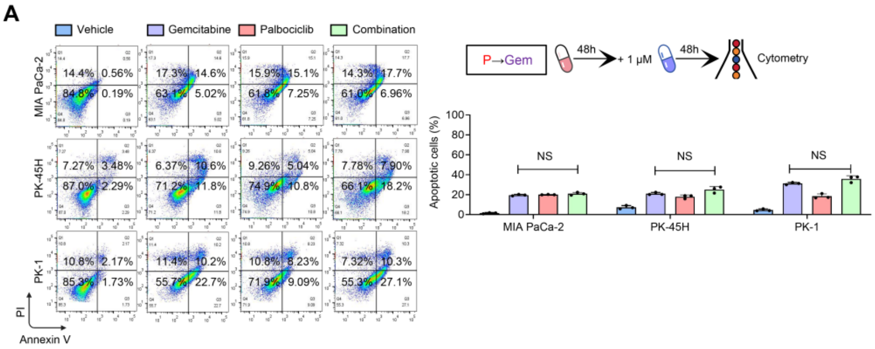

Supplementary Fig. 2 Palbociclib does not synergize with gemcitabine in PDAC. A Representative flow cytometry profiles of annexin V/PI double staining in the indicated cells pre-treated with or without 10 μM palbociclib for 48h thereafter with 1 μM gemcitabine for 48h. Quantitation of apoptotic cells (% = early apoptotic cells in Q2 + late apoptotic cells in Q3). DMSO was used as vehicle. All data are presented as mean ± SD of three independent experiments. One-way ANOVA followed by Tukey's post-hoc test was performed in **A**. *$p < 0.05$, **$p < 0.01$, ***$p < 0.001$.

Zhang et al., Supplementary Figure 3

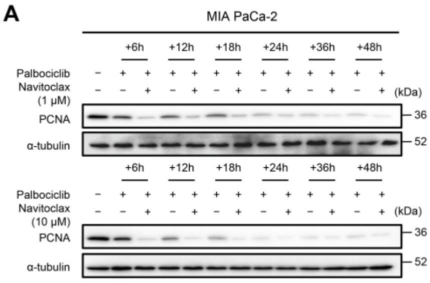

Supplementary Fig. 3 Senolytic agent enhances cell death in PDAC cells treated with CDK4/6 inhibitor without allowing re-entering to cell cycle. A IB of the indicated proteins in MIA PaCa-2 cells pre-treated with or without 10 μM palbociclib for 48h thereafter with the indicated doses of navitoclax for 0-48h. α-tubulin was used as a loading control.

Zhang et al., Supplementary Figure 4

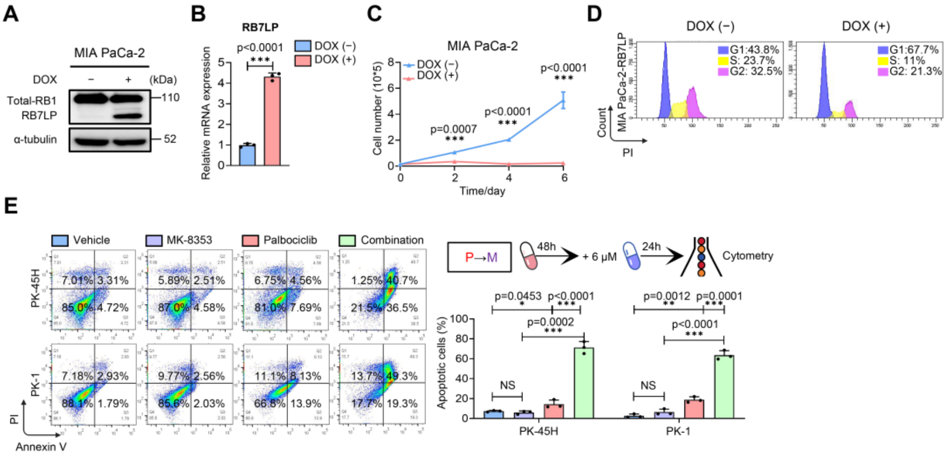

Supplementary Fig. 4 CDK4/6 inhibition sensitizes PDAC cells to ERK1/2 inhibitor. A IB of the indicated proteins in MIA PaCa-2-transduced with pTRE3G-puro-7LP-GFP (RB7LP) treated with or without 1 μg/mL DOX for 72h. **B** RT-qPCR determination of RB7LP in MIA PaCa-2-RB7LP cells treated as in (**A**). **C** Trypan blue-based assessment of growth property in MIA

PaCa-2-RB7LP cells treated with or without 1 μg/mL DOX for the indicated time. **D** Representative flow cytometry profiles of FxCycle™ PI/RNase staining in MIA PaCa-2-RB7LP cells treated as in (**A**). **E** Representative flow cytometry profiles of annexin V/PI double staining in the indicated cells pre-treated with or without 10 μM palbociclib for 48h thereafter with 6 μM MK-8353 for 24h. Quantitation of apoptotic cells (% = early apoptotic cells in Q2 + late apoptotic cells in Q3). α-tubulin was used as a loading control. DMSO was used as vehicle. All data are presented as mean ± SD of three independent experiments. Unpaired two-tailed Student's *t* test was performed in **B** and **C**. One-way ANOVA followed by Tukey's post-hoc test was performed in **E**. $^{*}p < 0.05$, $^{**}p < 0.01$, $^{***}p < 0.001$.

Zhang et al., Supplementary Figure 5

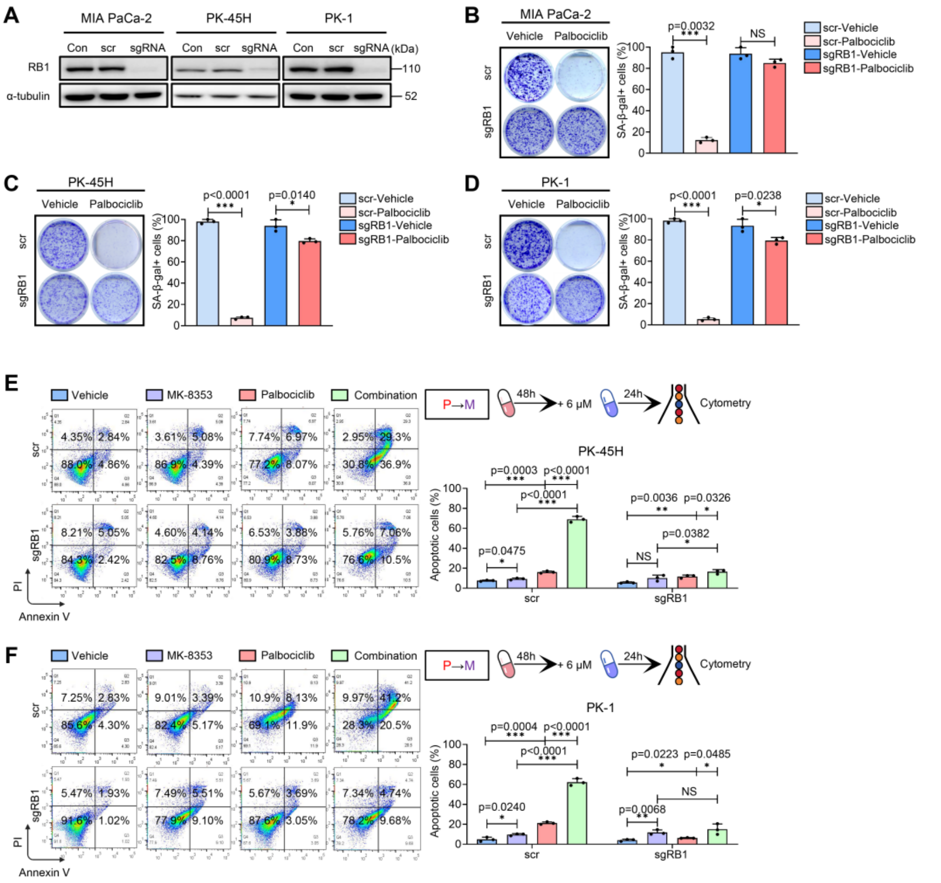

Supplementary Fig. 5 RB1 is necessary for the response to combination therapy with ERK inhibition. A IB of the indicated proteins in the indicated cells transduced with indicated vector. **B-D** Crstal violet staining of colonies from the indicated cell lines transduced with the indicated vector treated with or without 10 μM palbociclib. The relative colony number is normalized to vehicle (DMSO). **E-F** Representative flow cytometry profiles of annexin V/PI double staining in the indicated cells transduced with the indicated vector pre-treated with or without 10 μM palbociclib for 48h thereafter with 6 μM MK-8353 for 24h. Quantitation of apoptotic cells (% = early apoptotic cells in Q2 + late apoptotic cells in Q3). α-tubulin was used as a loading control. DMSO was used as

vehicle. All data are presented as mean ± SD of three independent experiments. Unpaired two-tailed Student's *t* test was performed in **B**-**D**. One-way ANOVA followed by Tukey's post-hoc test was performed in **E** and **F**. *$p < 0.05$, **$p < 0.01$, ***$p < 0.001$.

Zhang et al., Supplementary Figure 6

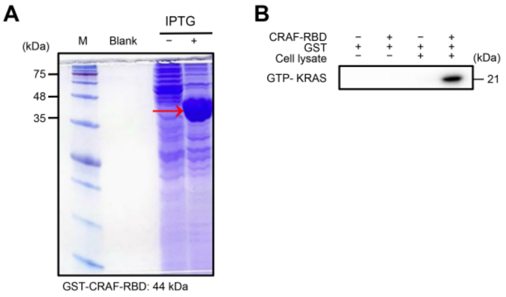

Supplementary Fig. 6 Purification of GST-CRAF-RBD. A Colloidal CBB staining of SDS-PAGE. **B** Pull down of GTP-KRAS using GST-CRAF-RBD proteins immobilized on glutathione-sepharose 4B. Bound proteins and cell lysates were analyzed by SDS-PAGE and following IB with anti-KRAS antibody.

Zhang et al., Supplementary Figure 7

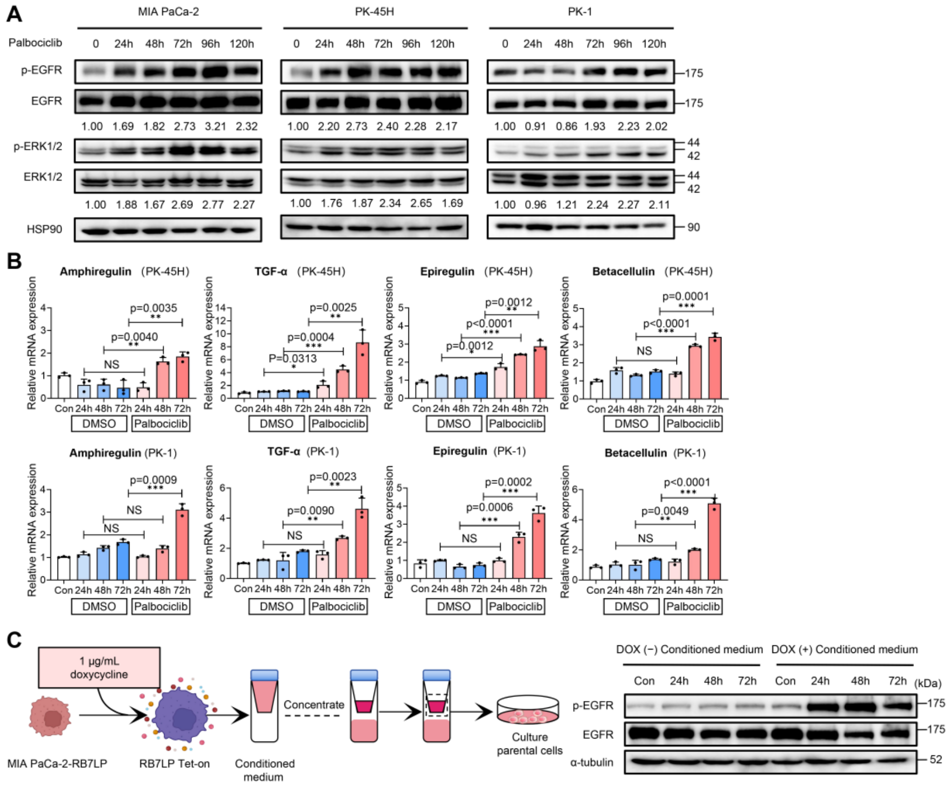

Supplementary Fig. 7 CDK4/6 inhibition induces SASP-mediated EGFR activation. A IB of the indicated proteins in the indicated cells treated with or without 10 μM palbociclib for 0-120h. **B** RT-qPCR determination of EGF family of ligands in the indicated cells treated with or without 10 μM palbociclib for 0-72h. **C** Schematic diagram of the procedure for the preparation of conditioned medium from MIA PaCa-2-RB7LP cells (left). IB of the indicated proteins in MIA PaCa-2 cells treated with DOX (−) or DOX (+) conditioned medium for 0-72h (right). α-tubulin was used as a loading control. DMSO was used as vehicle. All data are presented as mean ± SD of three independent experiments. One-way ANOVA followed by Tukey's post-hoc test was performed in **B**. *$p < 0.05$, **$p < 0.01$, ***$p < 0.001$.

Zhang et al., Supplementary Figure 8

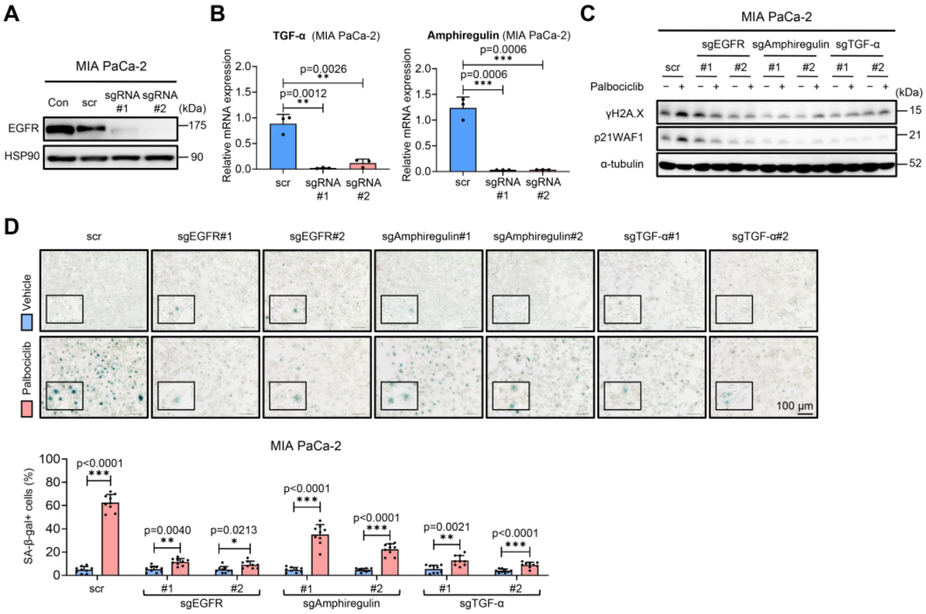

Supplementary Fig. 8 CDK4/6 inhibitor induces cellular senescence depending on EGFR ligands. A IB of the indicated proteins in MIA PaCa-2 cells transduced with the indicated vector. **B** RT-qPCR determination of TGF-α and Amphiregulin in MIA PaCa-2 cells transduced with the indicated vector. **C** IB of the indicated proteins in MIA PaCa-2 cells transduced with the indicated vector. **D** Representative images of SA-β-gal staining in MIA PaCa-2 cells transduced with the indicated vector treated with or without 10 μM palbociclib for 72h. Quantitation of SA-β-gal positive cells from 3 or more randomly chosen fields. HSP90 or α-tubulin was used as a loading control. DMSO was used as vehicle. All data are presented as mean ± SD of three independent experiments. One-way ANOVA followed by Tukey's post-hoc test was performed in **B**. Unpaired two-tailed Student's *t* test was performed in **D**. *$p < 0.05$, **$p < 0.01$, ***$p < 0.001$.

Zhang et al., Supplementary Figure 9

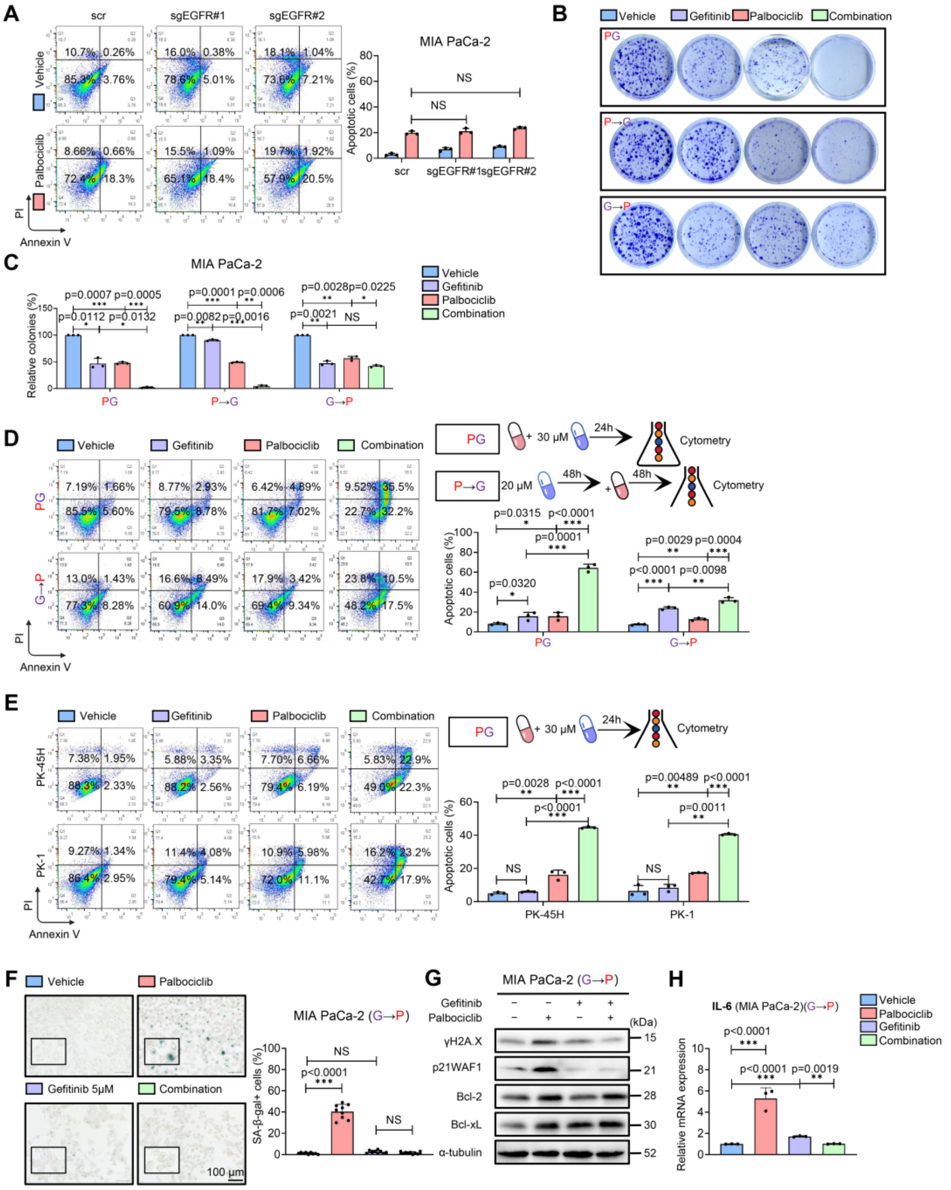

Supplementary Fig. 9 Sequence of treatment affects therapeutic efficacy

A Representative flow cytometry profiles of annexin V/PI double staining in the MIA PaCa-2 cells transduced with the indicated vector treated with or without 10 μM palbociclib for 72h. Quantitation of apoptotic cells (% = early apoptotic cells in Q2 + late apoptotic cells in Q3). **B**-**C** Crystal violet staining of colonies from MIA PaCa-2 cells. The relative colony number is normalized to vehicle (DMSO). PG: Cells were simultaneously treated with or without 1 μM

palbociclib and 5 μM gefitinib for 10 days. P→G: Cells were pre-treated with or without 1 μM palbociclib for 5 days thereafter with 5 μM gefitinib for 5 days. G→P: Cells were pre-treated with or without 5 μM gefitinib for 5 days thereafter with 1 μM palbociclib for 5 days. **D** Representative flow cytometry profiles of annexin V/PI double staining in MIA PaCa-2 cells treated with or without palbociclib and gefitinib. PG: Cells were simultaneously treated with or without 10 μM palbociclib and 30 μM gefitinib for 24h. G→P: Cells were pre-treated with or without 20 μM gefitinib for 48h thereafter with 10 μM palbociclib for 48h. Quantitation of apoptotic cells (% = early apoptotic cells in Q2 + late apoptotic cells in Q3). **E** Representative flow cytometry profiles of annexin V/PI double staining in PK-45H and PK-1 cells simultaneously treated with or without 10 μM palbociclib and 30 μM gefitinib for 24h. Quantitation of apoptotic cells (% = early apoptotic cells in Q2 + late apoptotic cells in Q3). **F** Representative images of SA-β-gal staining in MIA PaCa-2 cells pre-treated with or without 5 μM gefitinib for 48h thereafter with 10 μM palbociclib for 48h. Scale bars, 100 μm. Quantitation of SA-β-gal positive cells from 3 or more randomly chosen fields. **G** IB of the indicated proteins in MIA PaCa-2 cells treated as in (**F**). **H** RT-qPCR determination of IL-6 in MIA PaCa-2 cells treated as in (**F**). α-tubulin was used as a loading control. DMSO was used as vehicle. All data are presented as mean ± SD of three independent experiments. One-way ANOVA followed by Tukey's post-hoc test was performed in **A**, **C**, **D**, **E**, **F** and **H**. *$p < 0.05$, **$p < 0.01$, ***$p < 0.001$.

Zhang et al., Supplementary Figure 10

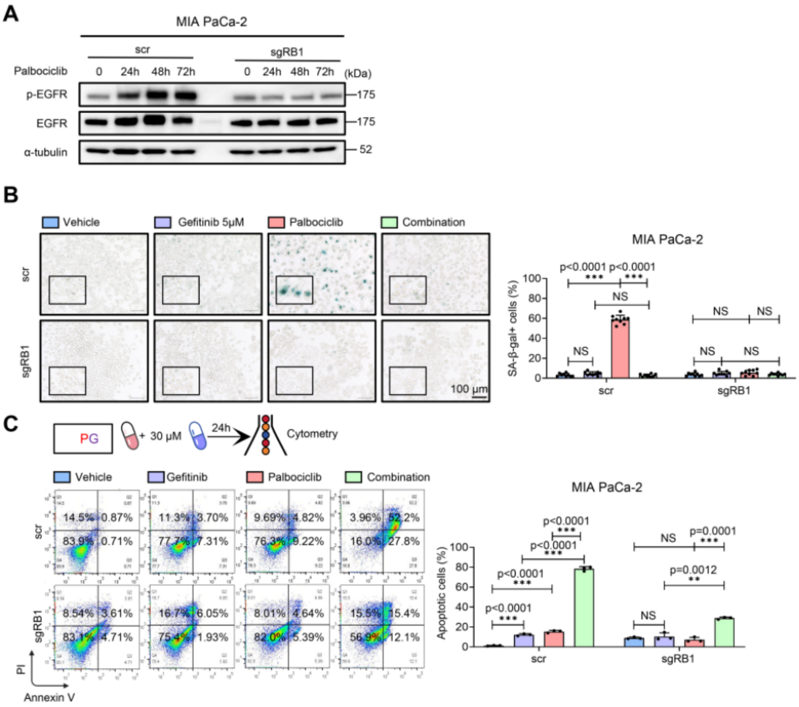

Supplementary Fig. 10 RB1 is necessary for the response to combination therapy. A IB of the indicated proteins in MIA PaCa-2-scr and MIA PaCa-2-sgRB1 cells treated with or without 10 μM palbociclib for 0-72h. **B** Representative images of SA-β-gal staining in the indicated cells pre-treated with or without 10 μM palbociclib for 48h thereafter with 5 μM gefitinib for 48h. Scale bars, 100 μm. Quantitation of SA-β-gal positive cells from 3 or more randomly chosen fields. **C** Representative flow cytometry profiles of annexin V/PI double staining in the indicated cells simultaneously treated with or without 10 μM palbociclib and 30 μM gefitinib for 24h. Quantitation of apoptotic cells (% = early apoptotic cells in Q2 + late apoptotic cells in Q3). α-tubulin was used as a loading control. DMSO was used as vehicle. All data are presented as mean ± SD of three independent experiments. One-way ANOVA followed

by Tukey's post-hoc test was performed in **B**, **C**. **p* < 0.05, ***p* < 0.01, ****p* < 0.001.

Zhang et al., Supplementary Figure 11

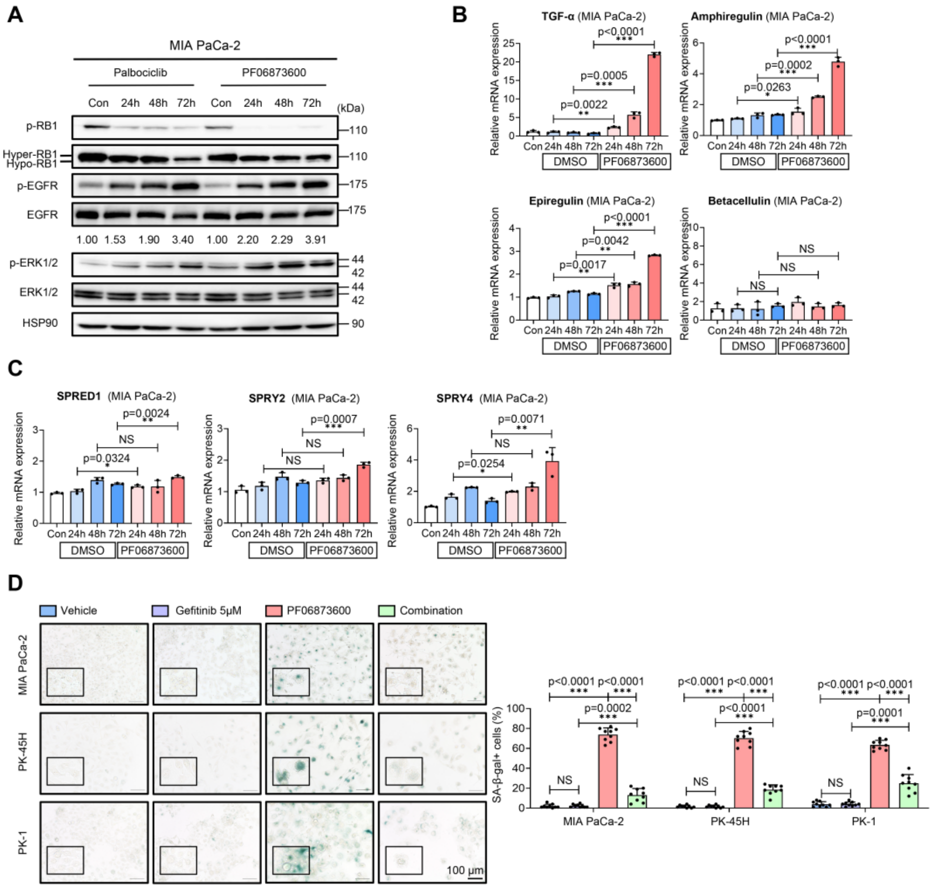

Supplementary Fig. 11 CDK2/4/6 inhibitor PF06873600 displays an efficacy comparable to palbociclib. A IB of the indicated proteins in MIA PaCa-2 cells treated with or without 10 μM palbociclib or 500 nM PF06873600 for 0-72h. **B-C** RT-qPCR determination of EGF family of ligands, Sprouty family members in MIA PaCa-2 cells treated with or without 500 nM PF06873600 for 0-72h. **D** Representative images of SA-β-gal staining in the indicated cells pre-treated with or without 500 nM PF06873600 for 48h thereafter with 5 μM gefitinib for 48h. Scale bars, 100 μm. Quantitation of

SA-β-gal positive cells from 3 or more randomly chosen fields. HSP90 was used as a loading control. DMSO was used as vehicle. All data are presented as mean ± SD of three independent experiments. One-way ANOVA followed by Tukey's post-hoc test was performed in **B**-**D**. **p* < 0.05, ***p* < 0.01, ****p* < 0.001.

Zhang et al., Supplementary Figure 12

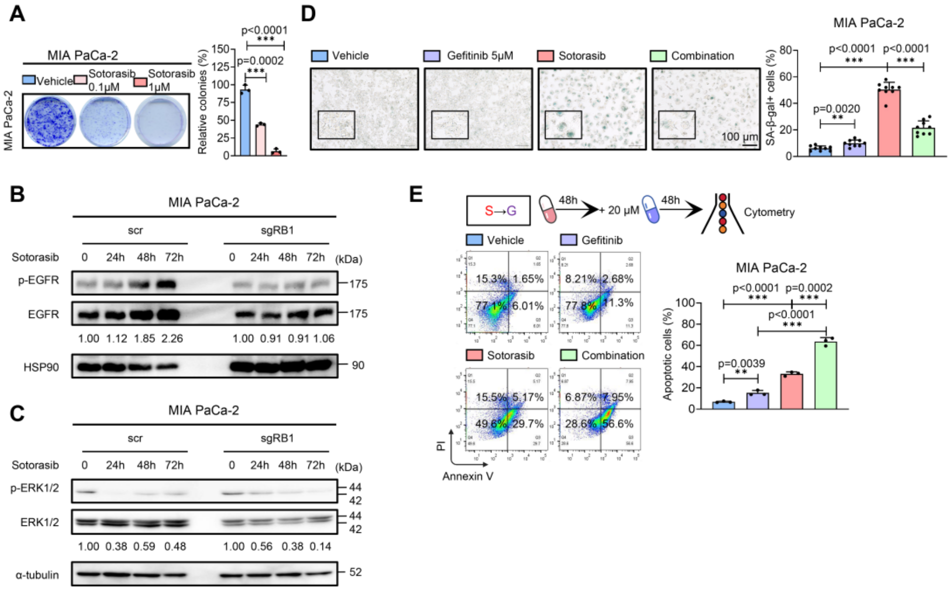

Supplementary Fig. 12 CDK4/6 and KRAS inhibitor commonly target many pathways to suppress PDAC. **A** Crystal violet staining of colonies from MIA PaCa-2 cells treated with or without indicated doses of sotorasib. The relative colony number is normalized to vehicle (DMSO). **B**-**C** IB of the indicated proteins in MIA PaCa-2 cells transduced with the indicated vector. **D** Representative images of SA-β-gal staining in MIA PaCa-2 cells pre-treated with or without 1 μM sotorasib for 48h thereafter with 5 μM gefitinib for 48h. Scale bars, 100 μm. Quantitation of SA-β-gal positive cells from 3 or more

randomly chosen fields. **E** Representative flow cytometry profiles of annexin V/PI double staining in the MIA PaCa-2 cells pre-treated with or without 1 μM sotorasib for 48h thereafter with 20 μM gefitinib for 48h. Quantitation of apoptotic cells (% = early apoptotic cells in Q2 + late apoptotic cells in Q3). HSP90 or α-tubulin was used as a loading control. DMSO was used as vehicle. All data are presented as mean ± SD of three independent experiments. One-way ANOVA followed by Tukey's post-hoc test was performed in **A**, **D** and **E**. *$p < 0.05$, **$p < 0.01$, ***$p < 0.001$.

Zhang et al., Supplementary Figure 13

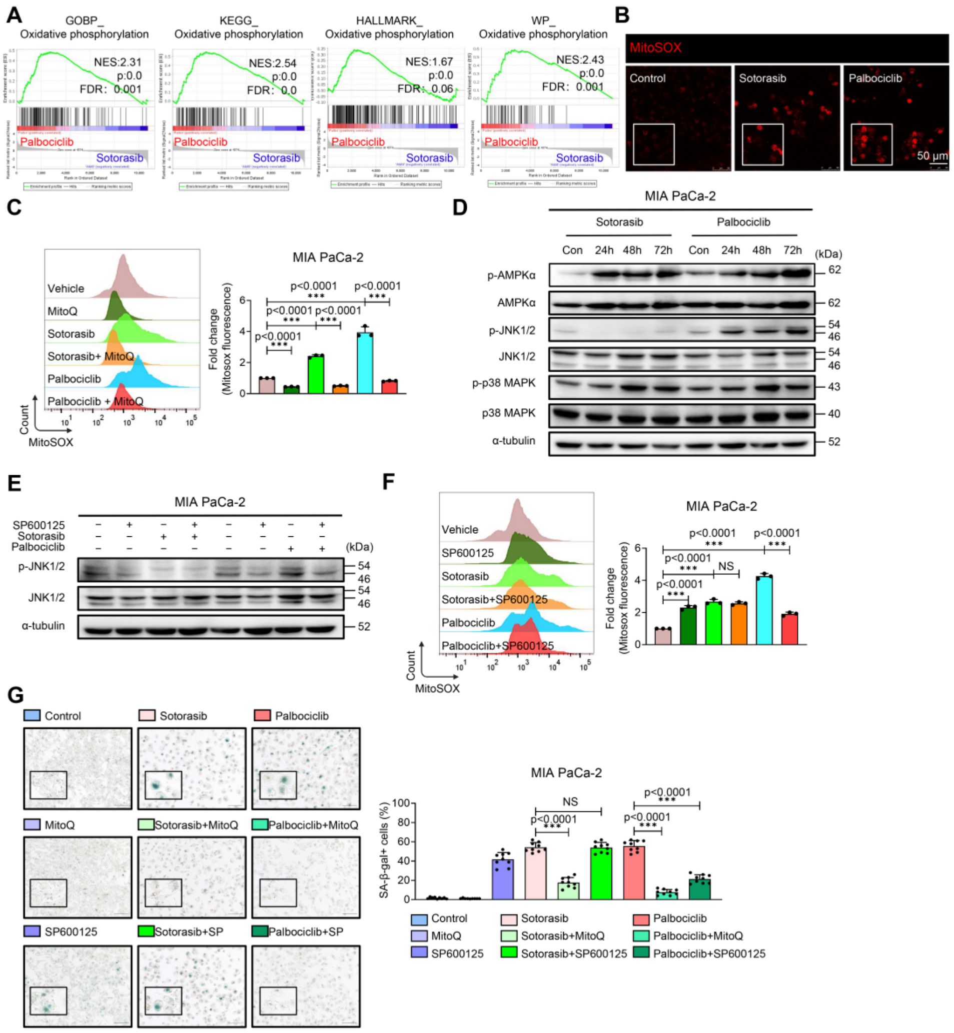

Supplementary Fig. 13 JNK mediates CDK4/6 inhibitor to induce cellular senescence. A Enrichment plots of OXPHOS pathway from GSEA provided by the indicated sources (MIA PaCa-2 cells). **B** Representative fluorescence microscopy images of MIA PaCa-2 cells treated with or without 1 μM sotorasib or 10 μM palbociclib for 72h stained with MitoSOX Red. Scale bars, 50 μm. **C** Representative flow cytometry histogram overlays of MitoSOX Red fluorescence intensity in MIA PaCa-2 cells simultaneously treated with or without 1 μM sotorasib, 10 μM palbociclib or 1 μM MitoQ for 72h. Fold change in mean fluorescence intensity were assessed. **D** IB of the indicated proteins in

MIA PaCa-2 cells treated with or without 1 μM sotorasib or 10 μM palbociclib for 0-72h. **E** IB of the indicated proteins in MIA PaCa-2 cells simultaneously treated with or without 1 μM sotorasib,10 μM palbociclib or 20 μM SP600125 for 72h. **F** Representative flow cytometry histogram overlays of MitoSOX Red fluorescence intensity in MIA PaCa-2 cells simultaneously treated with or without 1 μM sotorasib,10 μM palbociclib or 20 μM SP600125 for 72h. **G** Representative images of SA-β-gal staining in MIA PaCa-2 cells simultaneously treated with or without 1 μM sotorasib, 10 μM palbociclib, 1 μM MitoQ or 20 μM SP600125 for 72h. Quantitation of SA-β-gal positive cells from 3 or more randomly chosen fields. α-tubulin was used as a loading control. DMSO was used as vehicle. All data are presented as mean ± SD of three independent experiments. One-way ANOVA followed by Tukey's post-hoc test was performed in **C**, **F**, **G**. **p* < 0.05, ***p* < 0.01, ****p* < 0.001.

Zhang et al., Supplementary Figure 14

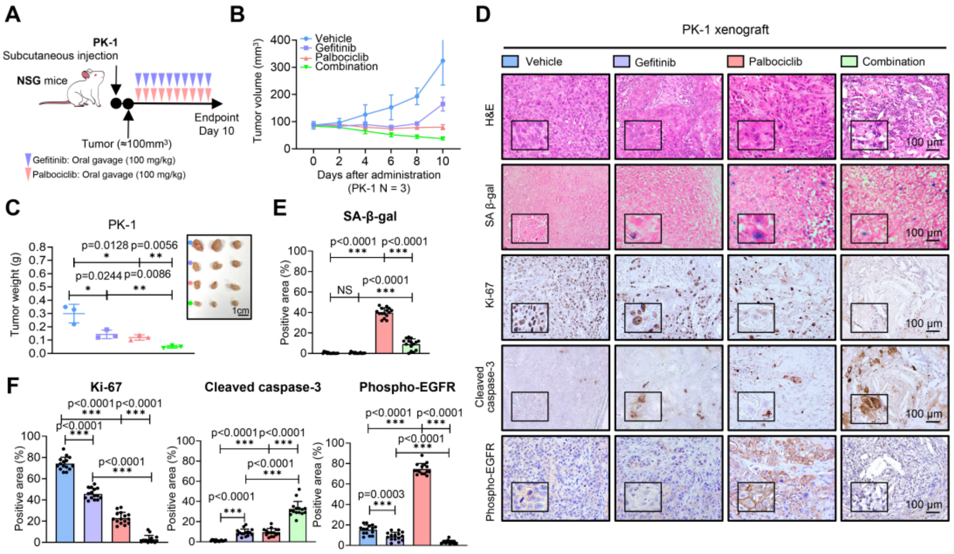

Supplementary Fig. 14 Simultaneuous inhibition of CDK4/6 and EGFR exhibits therapeutic efficacy in CDX model (PK-1). A Schematic diagram of experiments using NSG mice subcutaneously xenografted with PK-1 cells. Vehicle (corn oil or sodium L-lactate) or palbociclib (100 mg/kg, diluted in sodium L-lactate) together with gefitinib (100 mg/Kg, diluted in corn oil) were given daily via oral gavage and mice were euthanized at day 10 (N = 3). **B** Quantitation of volume of xenografts derived from NSG mice treated as in (**A**). Measurement of the tumor size were performed every 2 days. **C** Representative images and weights of xenografts at the endpoint. **D** SA-β-gal, H&E and IHC staining of xenografts derived from NSG mice treated as in (**A**). Scale bars, 100 μm. **E-F** Quantitation of SA-β-gal staining and immunostaining of the indicated proteins of xenografts derived from NSG mice treated as in (**A**). 5 randomly chosen fields observed per mouse were quantified under 20×microscope. All data are shown as mean ± SD of three independent experiments. One-way ANOVA followed by Tukey's post-hoc test was performed in **C**, **E** and **F**. *$p < 0.05$, **$p < 0.01$, ***$p < 0.001$.

Zhang et al., Supplementary Figure 15

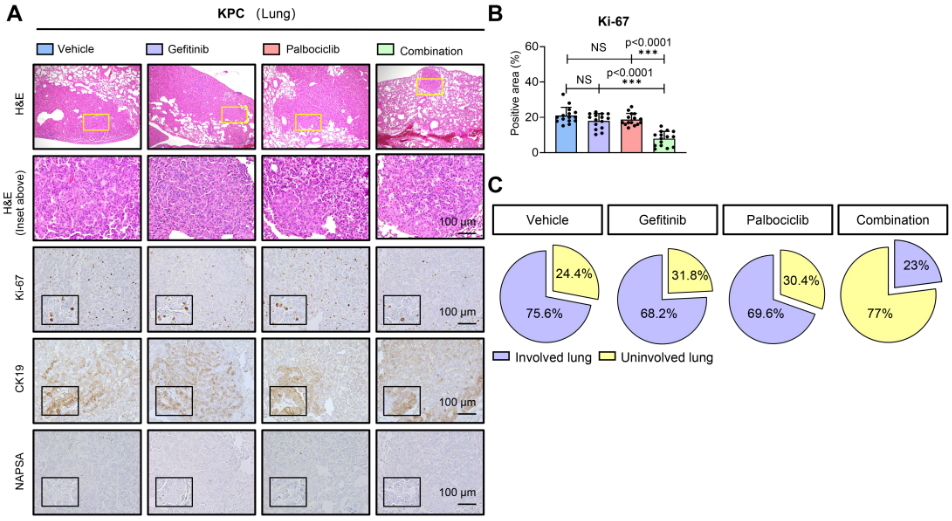

Supplementary Fig. 15 Combination therapy prevents lung metastasis. A H&E and IHC staining of lung in KPC mice treated as in Fig. 7**B**. Scale bars, 100 μm. **B** Quantitation of immunostaining of KPC mice treated as in Fig. 7**B**. 3 randomly chosen fields observed per mouse were quantified under 20×microscope. **C** Quantitation of relative percentage of tissue phenotype (N = 5). The average values were recorded. All data are presented as mean ± SD. One-way ANOVA followed by Tukey's post-hoc test was performed in **B**. *$p < 0.05$, **$p < 0.01$, ***$p < 0.001$.

Zhang et al., Supplementary Figure 16

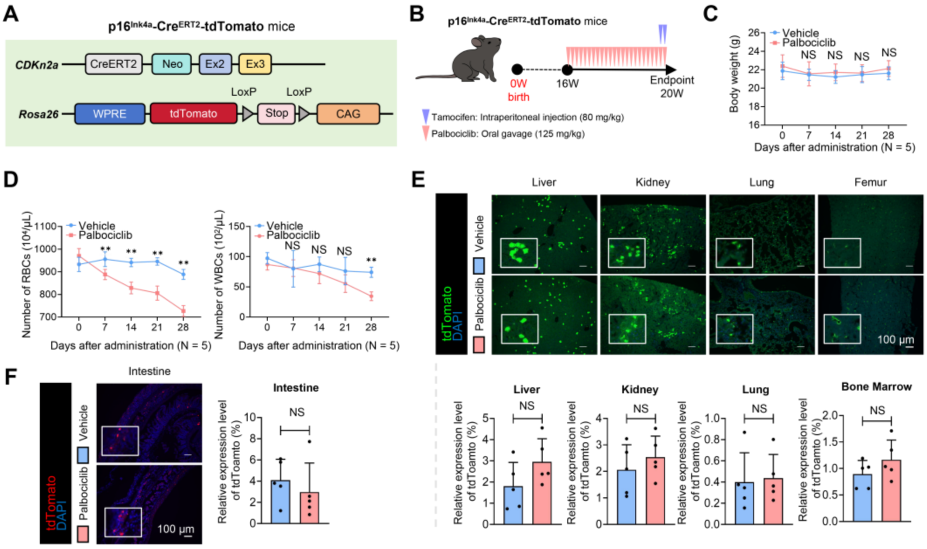

Supplementary Fig. 16 Therapeutic dose palbociclib does not induce p16^{Ink4a} in normal tissues. A Design of KPC mice model used in this study. **B** Schematic diagram of the experiments using p16^{Ink4a}-Cre^{ERT2}-tdTomato mice. Vehicle (sodium L-lactate) or palbociclib (125 mg/Kg, diluted in sodium L-lactate) were given via oral gavage for 4 weeks (5 days/week) (N = 5). Mice were euthanized after two intraperitoneal injections of tamoxifen (TAM, 80 mg/Kg). **C** Quantitation of body weight of mice treated as in (**B**). **D** Quantitation of RBCs and WBCs of mice treated as in (**B**). **E-F** Representative images of immunofluorescence staining for mice treated as in (**B**). Scale bar, 100 μm. Quantitation of fluorescence intensity in organs of mice were assessed. All data are presented as mean ± SD. Unpaired two-tailed Student's *t* test was performed in **C-F**. *$p < 0.05$, **$p < 0.01$, ***$p < 0.001$.
