# Supplementary material for: Deprivation of EGFR signal causes senolysis in PDAC with CDK4/6 inhibition
Source: Cell Death Differ. 2025 Dec 18;33(6):1218–33. doi: 10.1038/s41418-025-01634-0 (PMC13246951; doi:10.1038/s41418-025-01634-0)
Supplement: Supplementary file 2 — Supplementary TABLES [file 41418_2025_1634_MOESM2_ESM.pdf]

SUPPLEMENTARY TABLE

Deprivation of EGFR signal causes senolysis in PDAC with CDK4/6 inhibition

Yuanyuan Zhang[1], Susumu Kohno[1], Keqi Gao[2], Mahadi Hasan[3], Tomohisa Baba[2], Zixue Zhang[1,2], Nao Sankoda[4], Hai Yu [1], Junjian Pan [1], Noriko Gotoh[5], Makoto Nakanishi[6], Yasuhiro Yamada[4], Jindan Sheng[1,7,8,9], Takiko Daikoku[3], Yoshikazu Johmura[2] and Chiaki Takahashi[1,*]

[1]Division of Oncology and Molecular Biology, Cancer Research Institute, Kanazawa University, Kanazawa, Ishikawa 920-1192, Japan. [2]Division of Cancer and Senescence Biology, Cancer Research Institute, Kanazawa University, Kanazawa, Ishikawa 920-1192, Japan. [3]Division of Animal Disease Model, Research Center for Experimental Modeling of Human Disease, Kanazawa University, Kanazawa, Ishikawa 920-8640, Japan.[4]Department of Molecular Pathology, Graduate School of Medicine, The University of Tokyo, Bunkyo-ku, Tokyo 113-0033, Japan[5]Division of Cancer Cell Biology, Cancer Research Institute, Kanazawa University, Kanazawa, Ishikawa 920-1192, Japan.[6]Division of Cancer Cell Biology, Institute of Medical Science, The University of Tokyo, Tokyo, Minato-ku, 108-8639, Japan.[7]Maternal-Fetal Medicine and Gynecologic Oncology, Shanghai First Maternity and Infant Hospital, School of Medicine, Tongji University, Shanghai, 200092, China.[8]Department of Gynecology, Shanghai First Maternity and Infant Hospital, School of Medicine, Tongji University, Shanghai, 200092, China.

[9]Dana-Farber Cancer Institute, Harvard Medical School, Boston, MA 02215, USA.

* Corresponding author:

Chiaki Takahashi, Cancer Research Institute, Kanazawa University, Japan; Tel: +81-76-264-6750; Fax: +81-76-234-4521; E-mail: chtakaha@staff.kanazawa-u.ac.jp

Supplementary Table 1 The level of induction of SASP factors of MIA PaCa-2 cells following the indicated treatment.

SASP factors	Palbociclib (CDK 4/6 inhibitor)	Sotorasib ($KRAS^{G12C}$ inhibitor)
TGF-α	X12	X7
Amphiregulin	X18	X3
Epiregulin	X8	X1.5
Betacellulin	X5	X5
IL-6	X7	X7

Supplementary Table 2 The list of antibodies employed in this study.

Antibodies	Source	IDENTIFIER
Phospho-RB1	Cell Signaling Technology	Cat#8516, RRID:AB_11178658
RB1	Cell Signaling Technology	Cat#9313, RRID:AB_1904119
$p21^{WAF1}$	Cell Signaling Technology	Cat#2947, RRID:AB_823586
γH2A.X	Cell Signaling Technology	Cat#9718, RRID:AB_2118009
Phospho-CHK2	Cell Signaling Technology	Cat#2661, RRID:AB_331479
CHK2	Cell Signaling Technology	Cat#2662, RRID:AB_2080793
Bcl-2	Cell Signaling Technology	Cat#4223, RRID:AB_1903909
Bcl-xL	Cell Signaling Technology	Cat#2764, RRID:AB_2228008
PCNA	Cell Signaling Technology	Cat#13110, RRID:AB_2636979
PARP	Cell Signaling Technology	Cat#9542, RRID:AB_2160739
α-Tubulin	Cell Signaling Technology	Cat#3873, RRID:AB_1904178
RB7LP	Cell Signaling Technology	Cat#9309, RRID:AB_823629
Phospho-ERK1/2	Cell Signaling Technology	Cat#9101, RRID:AB_331646
ERK1/2	Cell Signaling Technology	Cat#9102, RRID:AB_330744
Phospho-EGFR	Cell Signaling Technology	Cat#3777, RRID:AB_2096270
EGFR	Cell Signaling Technology	Cat#4267, RRID:AB_2246311
Phospho-HER2/ErbB2	Cell Signaling Technology	Cat#2244, RRID:AB_331705
HER2/ErbB2	Cell Signaling Technology	Cat#2165, RRID:AB_10692490
Phospho-HER3/ErbB3	Cell Signaling Technology	Cat#4791, RRID:AB_2099709
Phospho-HER3/ErbB3	Millipore	Cat#05-390, RRID:AB_309713
Phospho-NF-κB	Cell Signaling Technology	Cat#3033, RRID:AB_331284
NF-κB	Cell Signaling Technology	Cat#8242, RRID:AB_10859369
Phospho-MEK1/2	Cell Signaling Technology	Cat#9121, RRID:AB_331648
MEK1/2	Cell Signaling Technology	Cat#9122, RRID:AB_823567
HSP90	Cell Signaling Technology	Cat#4874, RRID:AB_2121214
K-Ras	Cell Signaling Technology	Cat#33197
Pan-Ras	Millipore	Cat#MABS195, RRID:AB_3674178
Phospho-AMPKα	Cell Signaling Technology	Cat#2531, RRID:AB_330330
AMPKα	Cell Signaling Technology	Cat#5832, RRID:AB_10624867
Phospho-p38 MAPK	Cell Signaling Technology	Cat#9211, RRID:AB_331641
p38 MAPK	Cell Signaling Technology	Cat#9212, RRID:AB_330713
Phospho-SAPK/JNK	Cell Signaling Technology	Cat#9251, RRID:AB_331659
SAPK/JNK	Cell Signaling Technology	Cat#9252, RRID:AB_2250373
Ki-67	Cell Signaling Technology	Cat#12202, RRID:AB_2620142
Ki-67	Cell Signaling Technology	Cat#9027, RRID:AB_2636984
NAPSA	Proteintech	Cat#16558-1-AP, RRID:AB_2878278
RFP	MBL	Cat#PM005, RRID:AB_591279
CK19	Cell Signaling Technology	Cat#13092, RRID:AB_2722626
Cetuximab	Selleckchem	Cat#A2000, RRID:AB_2893090
Anti-Mouse IgG HRP	Cell Signaling Technology	Cat#7076, RRID:AB_330924
Anti-Rabbit IgG HRP	Cell Signaling Technology	Cat#7074, RRID:AB_2099233
Anti-Rabbit IgG Biotin	Jackson ImmunoResearch Labs	Cat#711-065-152, RRID:AB_2340593

Supplementary Table 2 The list of antibodies employed in this study (continued).

Antibodies	Source	IDENTIFIER
Anti-Mouse IgG Biotin	Thermo Fisher Scientific	Cat#31806, RRID:AB_228365
Anti-Mouse IgG HRP	VectorLabs	Cat#MP-7402, RRID:AB_2336528
Anti-Rabbit IgG HRP	VectorLabs	Cat#MP-7401, RRID:AB_2336529
Anti-Rabbit IgG Alexa Fluor ^{647}	Thermo Fisher Scientific	Cat#A-31573, RRID:AB_2536183
Anti-Rabbit IgG Alexa Fluor ^{488}	Thermo Fisher Scientific	Cat#A-11034, RRID:AB_2576217

Supplementary Table 3 The list of Taqman primers for RT-qPCR employed in this study.

Primer name	Assay ID	Source
Human-β actin	Hs99999903_m1	Thermo Fisher Scientific
Human-RB1	Hs01078066_m1	Thermo Fisher Scientific
Human-IL-6	Hs00985639_m1	Thermo Fisher Scientific

Supplementary Table 4 The list of primers for RT-qPCR employed in this study.

Primer name	Sequence (5'-3')
Human-TGF-α-FP	CCATTCTGGGTACGTTGGTG
Human-TGF-α-RP	CCACTCACAGTGTTTTCGGACC
Human-Amphiregulin-FP	GCACCTGGAAGCAGTAACATGC
Human-Amphiregulin-RP	GGCAGCTATGGCTGCTAATGCA
Human-Epiregulin-FP	CTTATCACAGTCGTCGGTTCCAC
Human-Epiregulin-RP	GACTTGCGGCAACTCTGGAT
Human-Betacellulin-FP	CCACCACACAATCAAAGCGG
Human-Betacellulin-RP	CTTTTACGACGTTTCCGAAGAGG
Human-SPRED1-FP	GAGACAGTTGTTACCAGTGAGCC
Human-SPRED1-RP	TGTCCAAGCCTGGCTGACCAAA
Human-SPRY2-FP	CCTGTTGCTGATGGCATAATCCG
Human-SPRY2-RP	ACTTGCCACAGTCCTCACACCT
Human-SPRY4-FP	AGGTGAAGACCAGCCATGTGGA
Human-SPRY4-RP	TCCAATGGTGGGTGACATCCTG

Supplementary Table 5 The list of GRISPR/Cas9 sgRNA primers employed in this study.

GRISPR/Cas9 sgRNA primer	Sequence (5'-3')
Human RB1 sgRNA	TAGGCTAGCCGATACACTGT
Human EGFR sgRNA1	TGTCACCACATAATTACCTG
Human EGFR sgRNA2	GAGAACCTAGAAATCATACG
Human TGF-α sgRNA1	GTGCACCAACGTACCCAGAA
Human TGF-α sgRNA2	GATGGAGACCACCACCAAGG
Human Amphiregulin sgRNA1	GTGCTGTCGCTCTTGATACT
Human Amphiregulin sgRNA2	TCATAGTCGGCTCCCGAGGA

Supplementary Table 6 The list of primers for mice genotyping.

Genotyping primer	Sequence (5'-3')	
Pdx1-ires-Cre	Fw	AGAGCCGGAGCAAGATTGT
	Rv1	TCAGAAGCTCAGGGCTGTTT
	Rv2	GTTGCATCGACCGGTAATGC
LSL-Kras^{G12D}	Fw	CTAGCCACCATGGCTTGAGT
	Rv	TCCGAATTCAGTGACTACAGATG
LSL-Trp53^{R172H}	FW	AGGTGTGGCTTCTGGCTT C
	Rv1	GAAACTTTTCACAAGAACCAGATCA
	Rv2	CCAGCTCATTCCTCCCACTC
p16^{Ink4a}*-Cre*^{ERT2}	FW	GTTTCACTGGTTATGCGGCGG
	Rv	TTCCAGGGCGCGAGTTGATA

Supplementary Table 7 Chemical compounds used in this study.

Chemical compounds	Source	IUPAC Name
Palbociclib (PD-0332991)	LC laboratory	6-acetyl-8-cyclopentyl-5-methyl-2-[(5-piperazin-1-ylpyridin-2-yl)amino]pyrido[2,3-d]pyrimidin-7-one
Navitoclax (ABT-263)	Selleckchem	4-[4-[[2-(4-chlorophenyl)-5,5-dimethylcyclohexen-1-yl]methyl]piperazin-1-yl]-N-[4-[[(2R)-4-morpholin-4-yl-1-phenylsulfanylbutan-2-yl]amino]-3-(trifluoromethylsulfonyl)phenyl]sulfonylbenzamide
Gemcitabine (LY-188011)	Selleckchem	4-amino-1-[(2R,4R,5R)-3,3-difluoro-4-hydroxy-5-(hydroxymethyl)oxolan-2-yl]pyrimidin-2-one
MK-8353 (SCH900353)	Selleckchem	(3S)-3-methylsulfanyl-1-[2-[4-[4-(1-methyl-1,2,4-triazol-3-yl)phenyl]-3,6-dihydro-2H-pyridin-1-yl]-2-oxoethyl]-N-[3-(6-propan-2-yloxypyridin-3-yl)-1H-indazol-5-yl]pyrrolidine-3-carboxamide
Z-VAD-FMK (Z-VAD(OMe)-FMK)	Peptide Institute, Inc.	methyl(3S)-5-fluoro-3-[[(2S)-2-[[(2S)-3-methyl-2-(phenylmethoxycarbonylamino)butanoyl]amino]propanoyl]amino]-4-oxopentanoate
Gefitinib (G-4408)	LC laboratory	N-(3-chloro-4-fluorophenyl)-7-methoxy-6-(3-morpholin-4-ylpropoxy)quinazolin-4-amine
PF06873600 (Ebvaciclib)	Selleckchem	6-(difluoromethyl)-8-[(1R,2R)-2-hydroxy-2-methylcyclopentyl]-2-[(1-methylsulfonylpiperidin-4-yl)amino]pyrido[2,3-d]pyrimidin-7-one
Sotorasib (AMG-510)	Selleckchem	6-fluoro-7-(2-fluoro-6-hydroxyphenyl)-1-(4-methyl-2-propan-2-ylpyridin-3-yl)-4-[(2S)-2-methyl-4-prop-2-enoylpiperazin-1-yl]pyrido[2,3-d]pyrimidin-2-one
JSH-23	Selleckchem	4-methyl-N~1~-(3-phenylpropyl)-1,2-benzenediamine
Mitoquinone (MitoQ)	Selleckchem	10-(4,5-dimethoxy-2-methyl-3,6-dioxocyclohexa-1,4-dien-1-yl)decyl-triphenylphosphanium
SP600125 (Nsc75890)	Selleckchem	14,15-diazatetracyclo[7.6.1.0^{2,7}.0^{13,16}]hexadeca-1(15),2,4,6,9(16),10,12-heptaen-8-one
IPTG	Nacalai tesque	Propan-2-yl 1-thio-β-D-galactopyranoside
Tamocifen	Selleckchem	2-{4-[(1Z)-1,2-diphenylbut-1-en-1-yl]phenoxy}-N,N-dimethylethanamine
