# Supplementary material for: Deprivation of EGFR signal causes senolysis in PDAC with CDK4/6 inhibition
Source: Cell Death Differ. 2025 Dec 18;33(6):1218–33. doi: 10.1038/s41418-025-01634-0 (PMC13246951; doi:10.1038/s41418-025-01634-0)

Supplementary DATA UNCROPPED IMMUNOBLOIING

Deprivation of EGFR signal causes senolysis in PDAC with CDK4/6 inhibition

Yuanyuan Zhang[1], Susumu Kohno[1], Keqi Gao[2], Mahadi Hasan[3], Tomohisa Baba[2], Zixue Zhang[1,2], Nao Sankoda[4], Hai Yu [1], Junjian Pan [1], Noriko Gotoh[5], Makoto Nakanishi[6], Yasuhiro Yamada[4], Jindan Sheng[1,7,8,9], Takiko Daikoku[3], Yoshikazu Johmura[2] and Chiaki Takahashi[1,*]

[1]Division of Oncology and Molecular Biology, Cancer Research Institute, Kanazawa University, Kanazawa, Ishikawa 920-1192, Japan. [2]Division of Cancer and Senescence Biology, Cancer Research Institute, Kanazawa University, Kanazawa, Ishikawa 920-1192, Japan. [3]Division of Animal Disease Model, Research Center for Experimental Modeling of Human Disease, Kanazawa University, Kanazawa, Ishikawa 920-8640, Japan.[4]Department of Molecular Pathology, Graduate School of Medicine, The University of Tokyo, Bunkyo-ku, Tokyo 113-0033, Japan[5]Division of Cancer Cell Biology, Cancer Research Institute, Kanazawa University, Kanazawa, Ishikawa 920-1192, Japan.[6]Division of Cancer Cell Biology, Institute of Medical Science, The University of Tokyo, Tokyo, Minato-ku, 108-8639, Japan.[7]Maternal-Fetal Medicine and Gynecologic Oncology, Shanghai First Maternity and Infant Hospital, School of Medicine, Tongji University, Shanghai, 200092, China.[8]Department of Gynecology, Shanghai First Maternity and Infant Hospital, School of Medicine, Tongji University, Shanghai, 200092, China.

[9]Dana-Farber Cancer Institute, Harvard Medical School, Boston, MA 02215, USA.

* Corresponding author:

Chiaki Takahashi, Cancer Research Institute, Kanazawa University, Japan; Tel: +81-76-264-6750; Fax: +81-76-234-4521; E-mail: chtakaha@staff.kanazawa-u.ac.jp

UNCROPPED IMMUNOBLOIINGS

Fig.1B

MIA PaCa-2 (left)

p-RB1(0-72h)

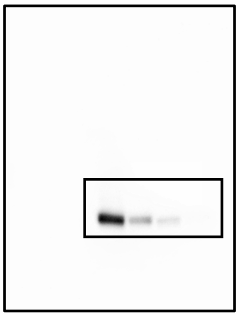

RB1(0-72h)

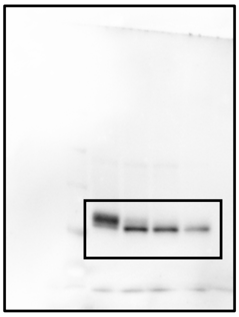

HSP90(0-72h)

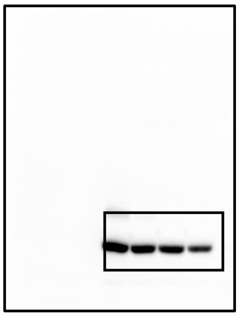

p21WAF1(0-72h)

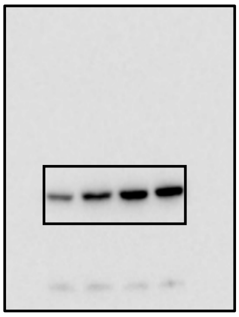

p-γH2AX(0-72h)

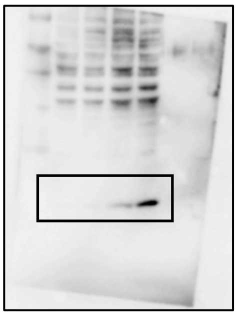

p-CHK2(0-72h)

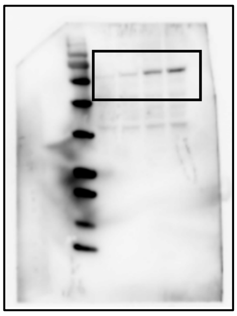

CHK2 (0-72h)

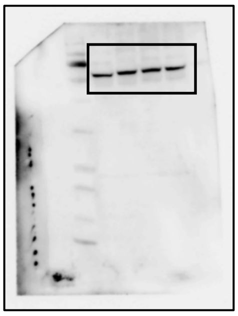

Cleaved-PARP(0-72h)

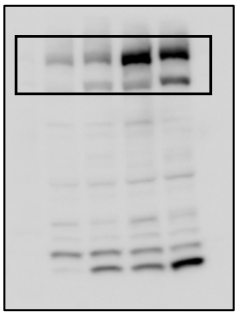

α-tubulin(72h)

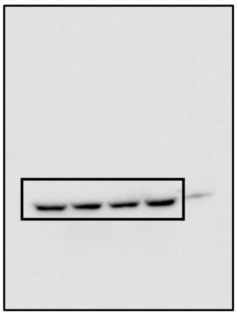

Bcl-2(0-120h)

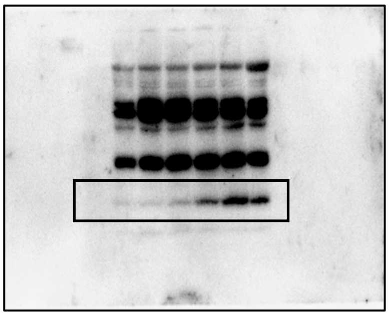

Bcl-xL(0-120h)

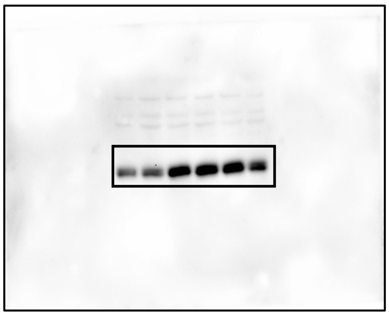

PCNA(0-120h) α-tubulin(0-120h)

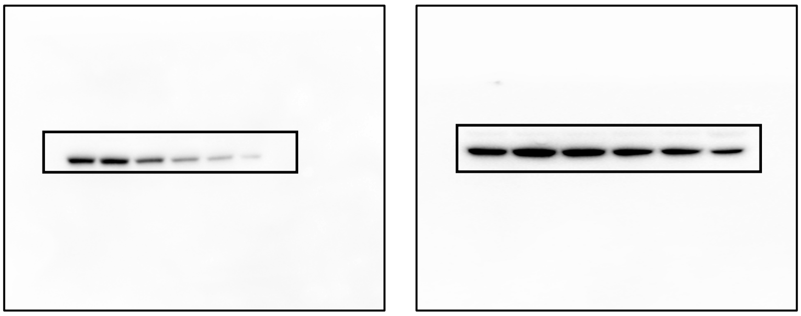

PK-45H (middle)

p-RB1 RB1 p21WAF1 γH2A.X p-CHK2 CHK2

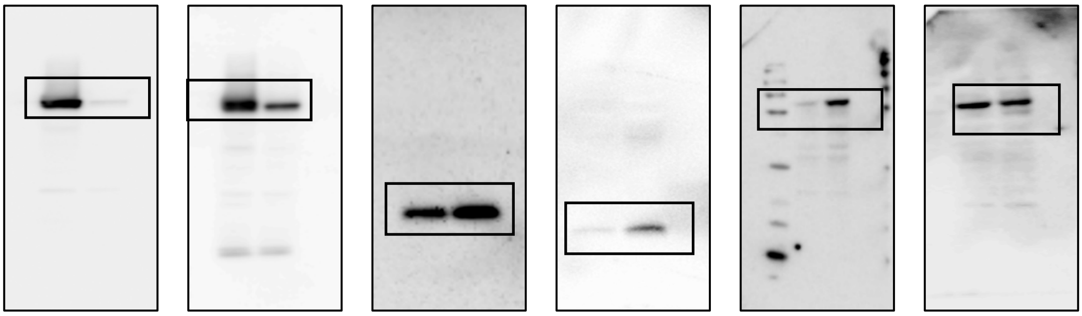

Bcl-2 Bcl-xL PCNA Cleaved-PARP α-tubulin

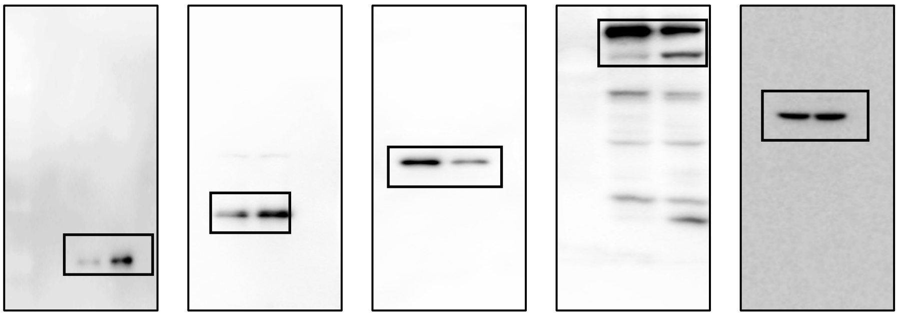

PK-1 (right)

p-RB1 RB1 p21WAF1 γH2A.X p-CHK2

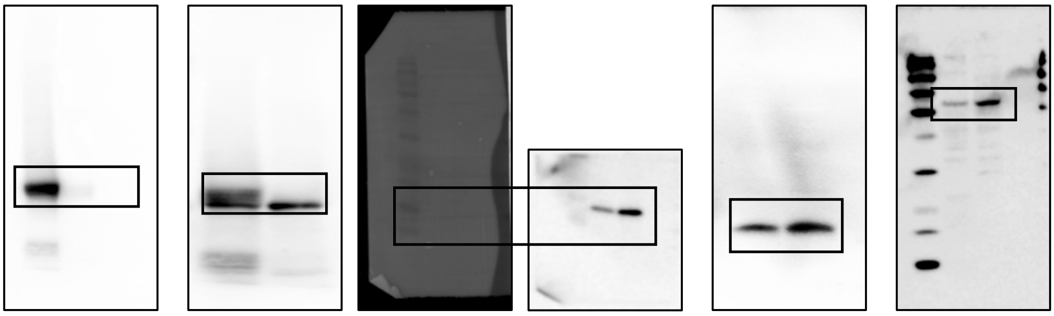

CHK2

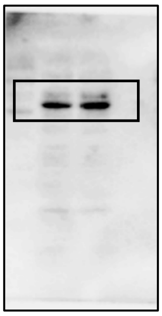

Bcl-2

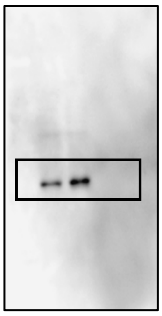

Bcl-xL

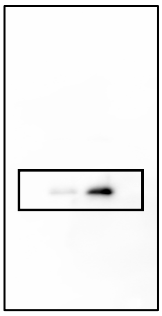

PCNA

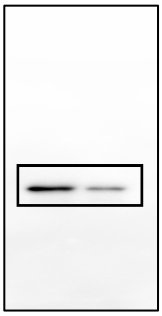

Cleaved-PARP

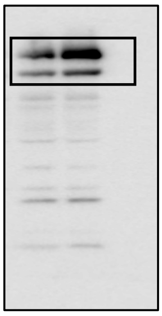

α-tubulin

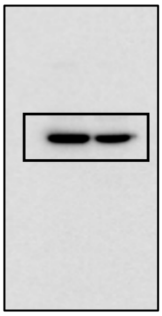

Fig.1H

PCNA

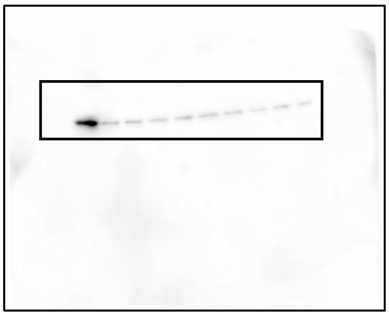

p21WAF1

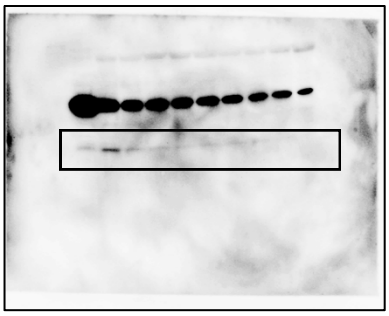

Cleaved-PARP

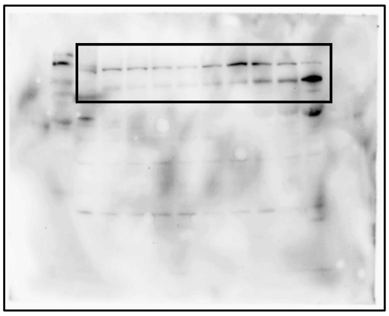

Bcl-2

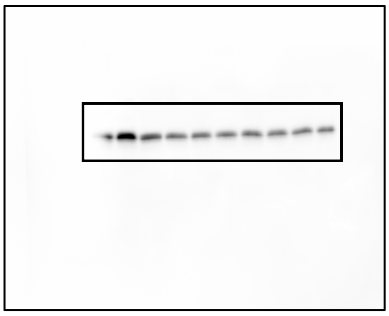

α-tubulin

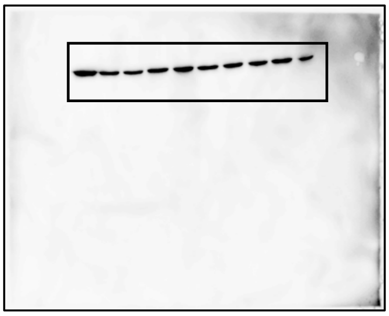

Fig.2B

p21WAF1 γH2A.X p-CHK2 CHK2

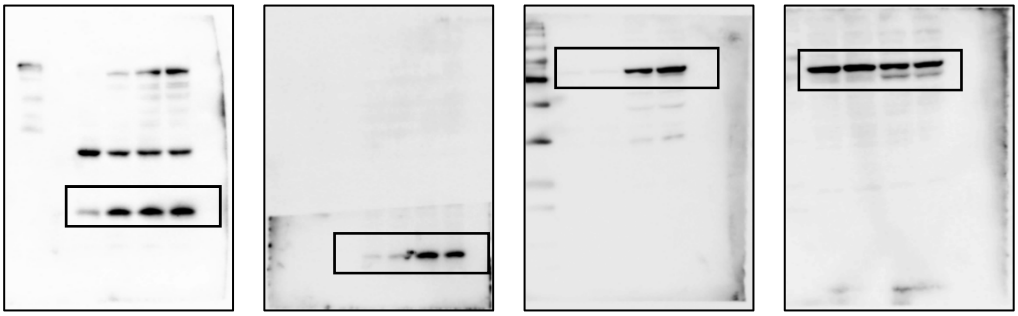

Bcl-2 PCNA Cleaved-PARP α-tubulin

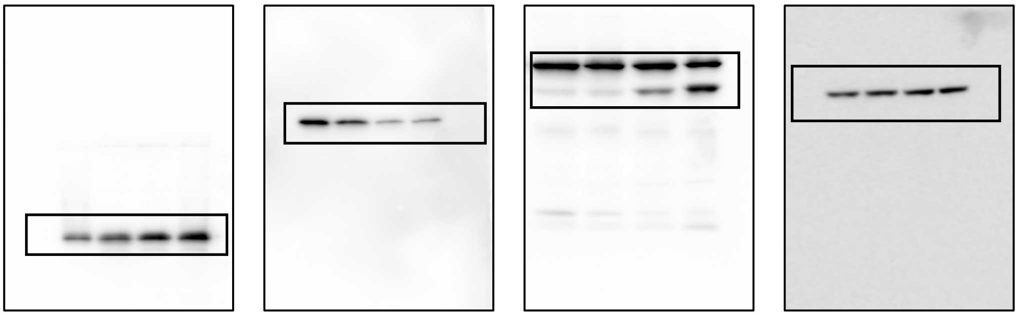

Fig.2G

p-ERK1/2 ERK1/2 α-tubulin

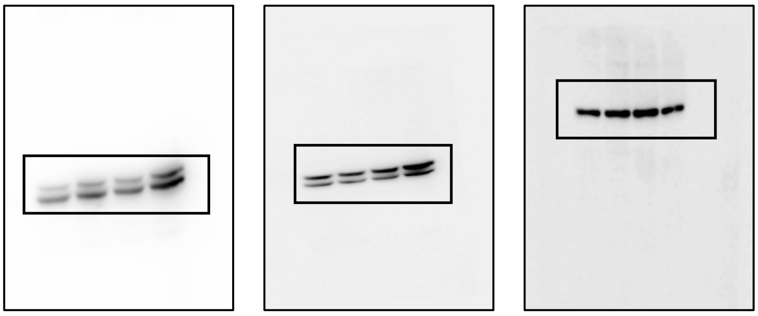

Fig.2H

p-ERK1/2 ERK1/2 Cleaved-PARP α-tubulin

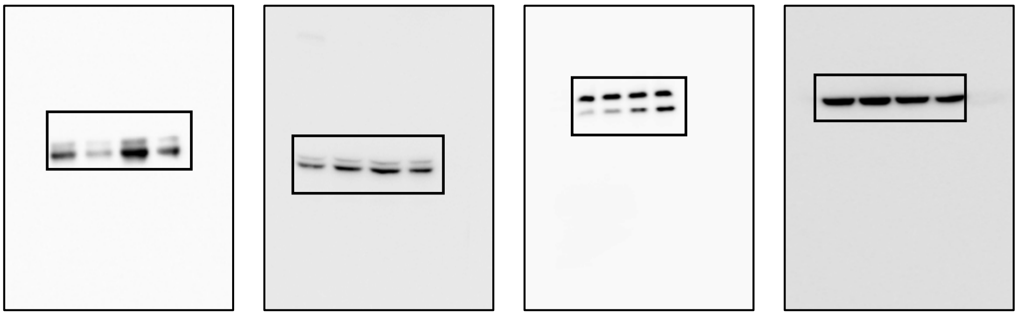

Fig.2J

p-ERK1/2(0-96h) ERK1/2(0-96h) α-tubulin(0-96h)

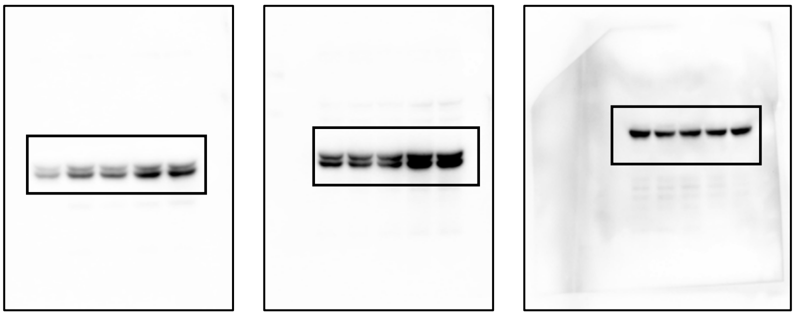

Fig.2K

p-ERK1/2 ERK1/2 Cleaved-PARP α-tubulin

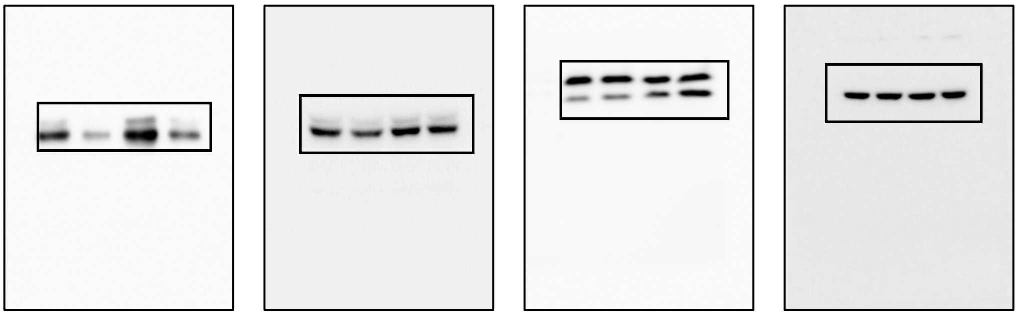

Fig.2N

p-ERK1/2 ERK1/2 α-tubulin

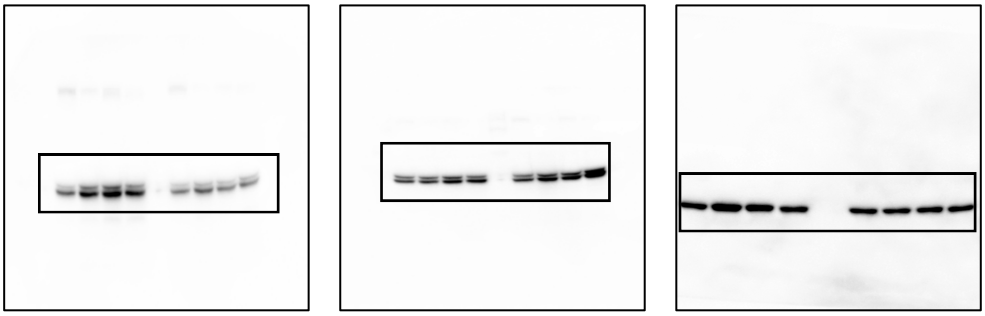

Fig.3A

GTP-KRAS KRAS α-tubulin

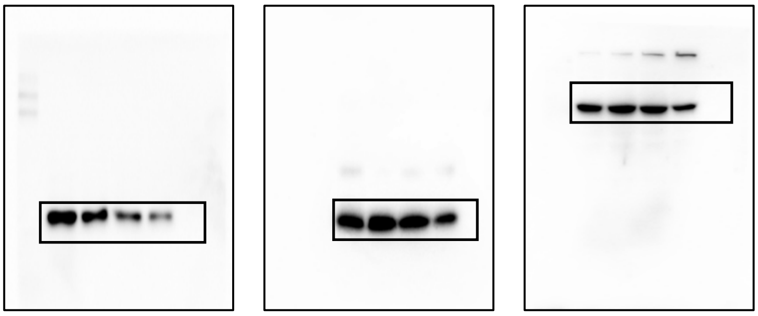

Fig.3B

GTP-KRAS KRAS GTP-Pan-RAS Pan-RAS

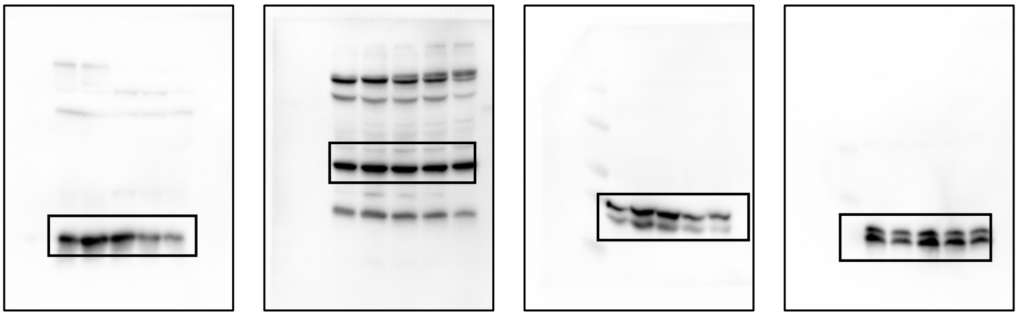

α-tubulin

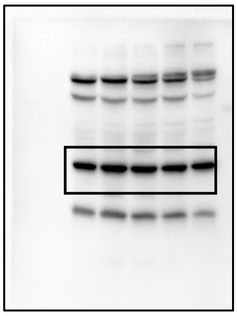

Fig.3C

p-EGFR EGFR p-HER2/ErbB2 HER2/ErbB2

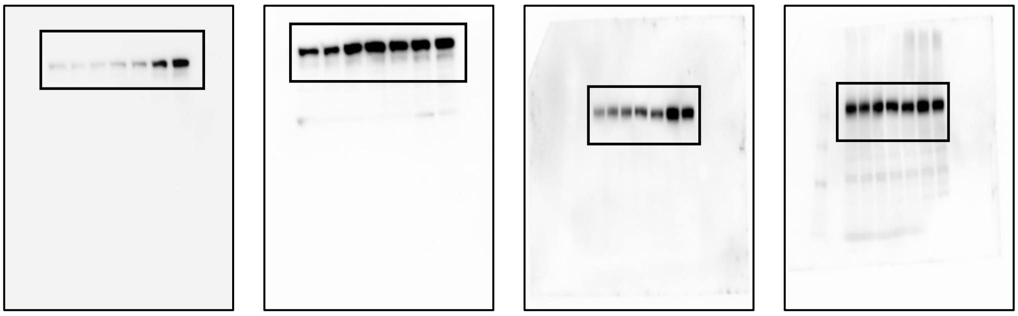

p-HER3/ErbB3 HER3/ErbB3 p-ERK1/2 ERK1/2

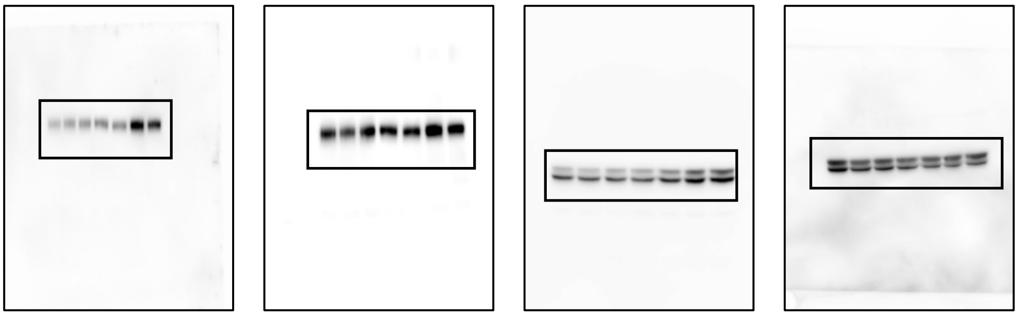

p21WAF1

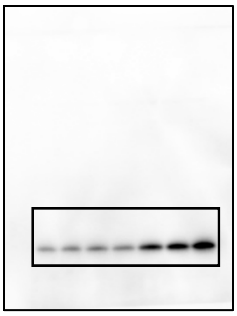

α-tubulin

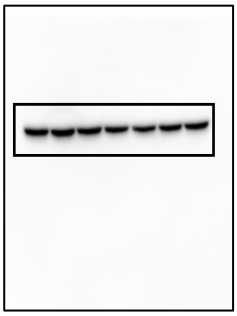

Fig.3H

p-NF-κB

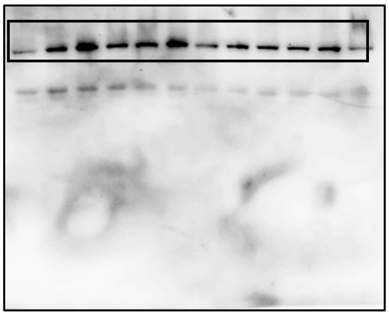

NF-κB

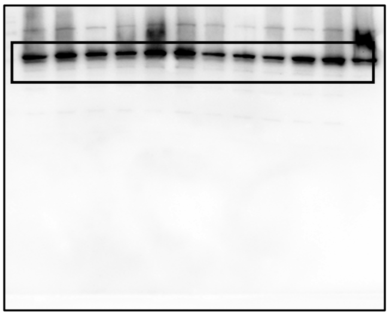

Bcl-2

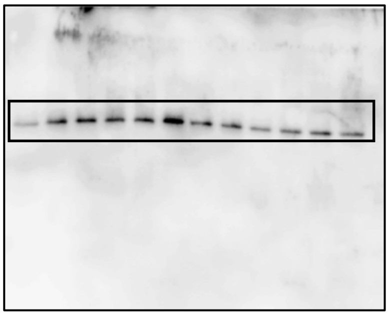

p21WAF1

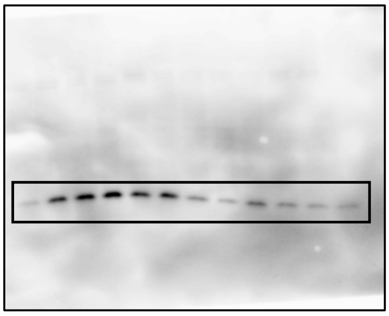

PCNA

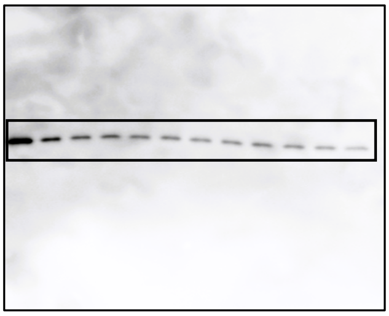

p-EGFR

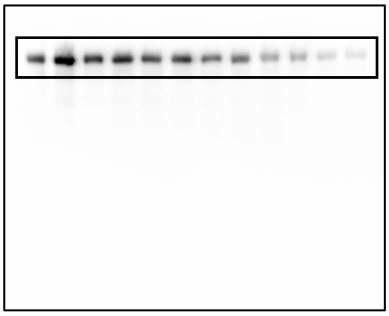

EGFR

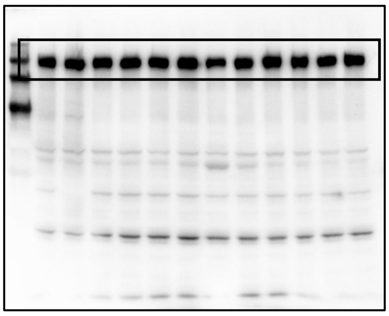

α-tubulin

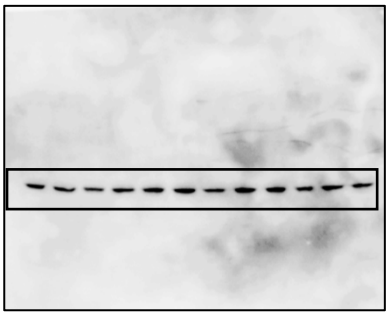

Fig.4A

p-EGFR

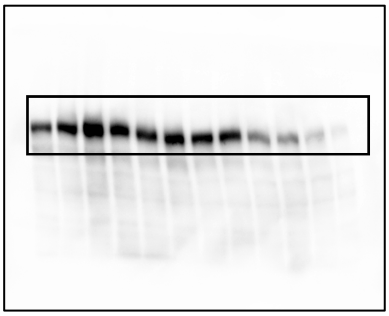

EGFR

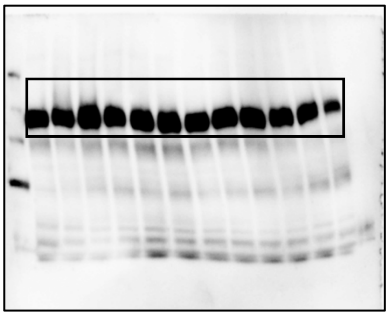

Bcl-2

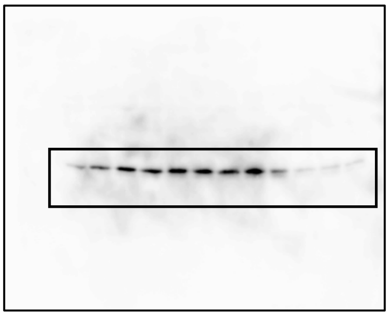

Bcl-xL

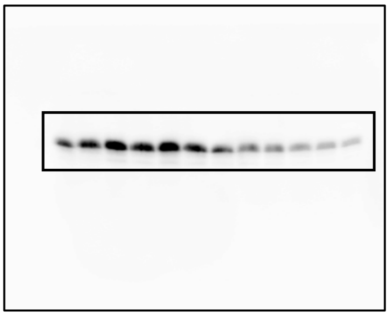

PCNA

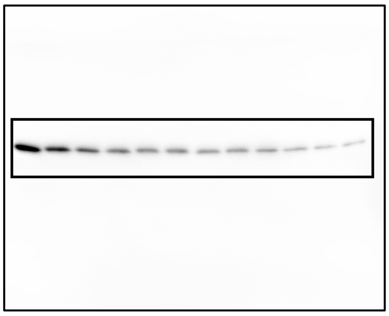

α-tubulin

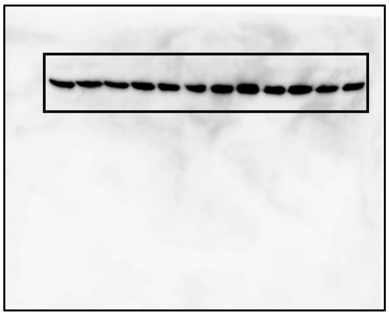

Fig.4C

PCNA(top)

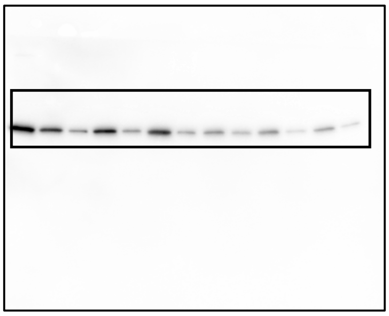

α-tubulin(top)

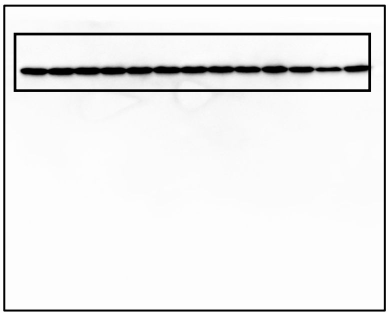

PCNA(bottom)

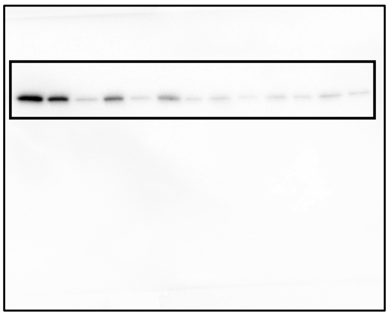

α-tubulin(bottom)

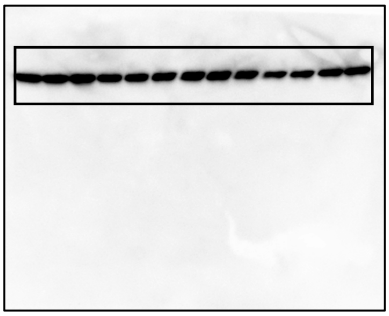

Fig.4D

p21WAF1

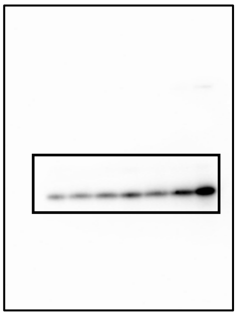

γH2A.X

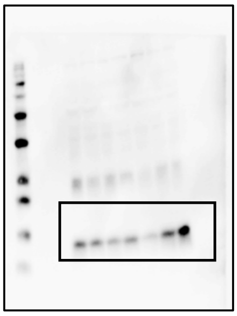

Bcl-2

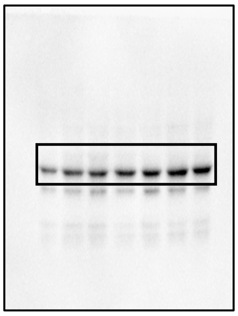

Bcl-xL

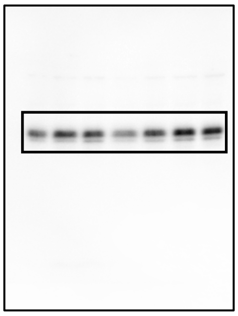

PCNA

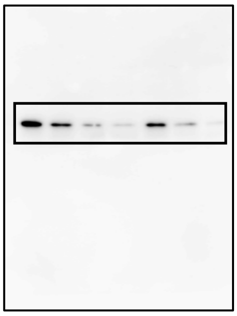

p-EGFR

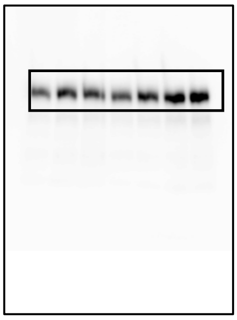

EGFR

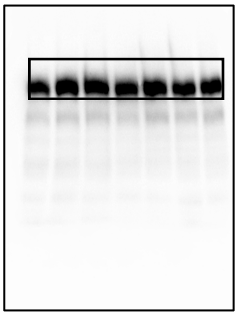

p-ERK1/2

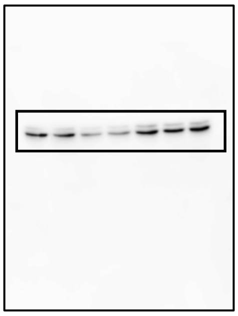

ERK1/2 α-tubulin

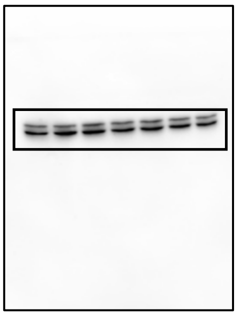
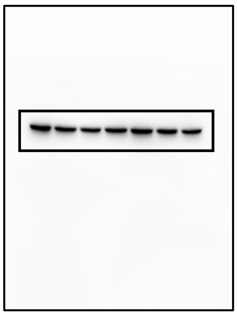

Fig.5A

p-RB1 RB1 p-EGFR EGFR

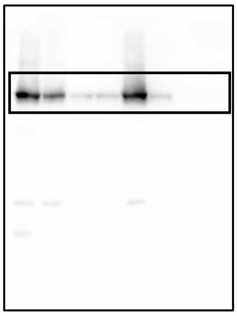
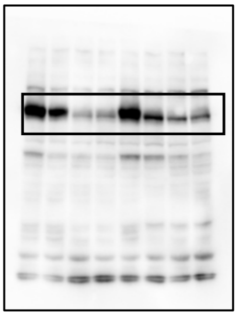
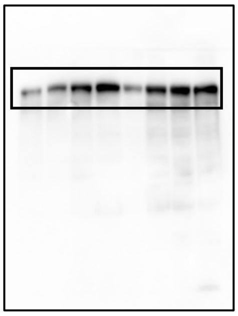
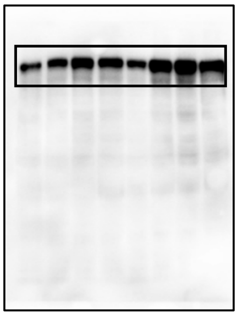

p-MEK1/2 MEK p-ERK1/2 ERK1/2

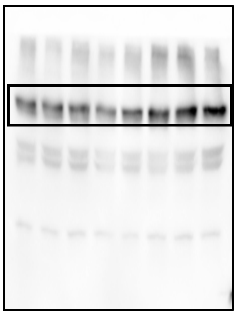
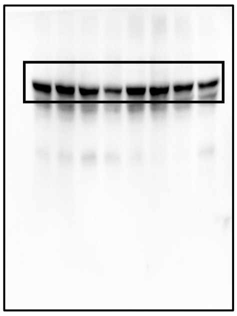
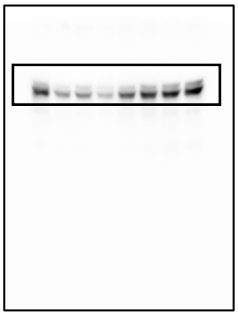
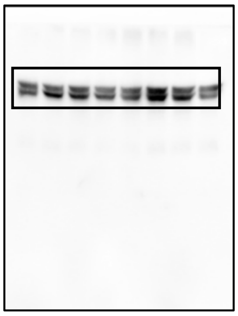

p-NF-κB NF-κB p21WAF1 Bcl-2

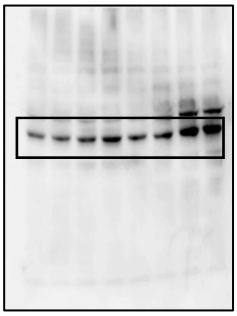
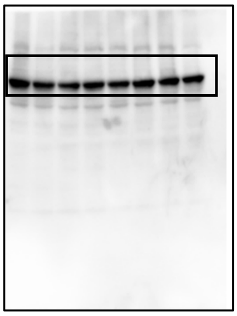
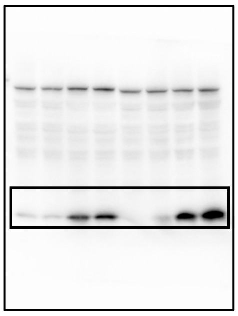
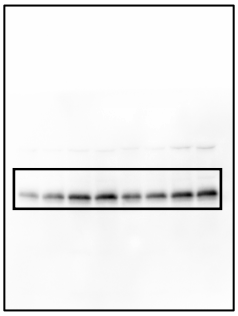

α-tubulin

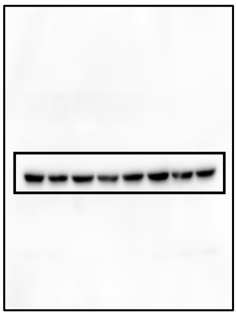

Fig.5B

GTP-KRAS KRAS α-tubulin

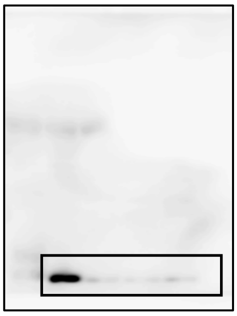

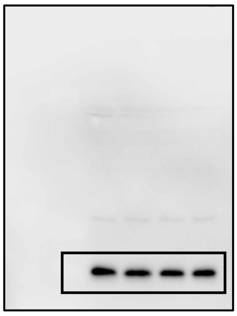

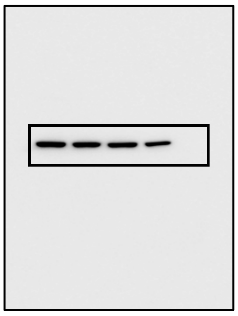

Supplementary Fig.3A

PCNA(up) α-tubulin(upper)

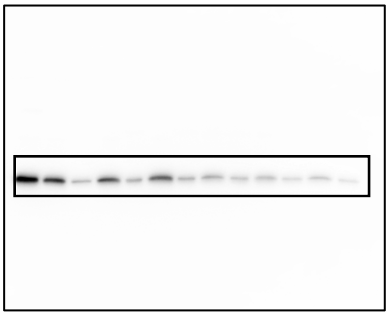

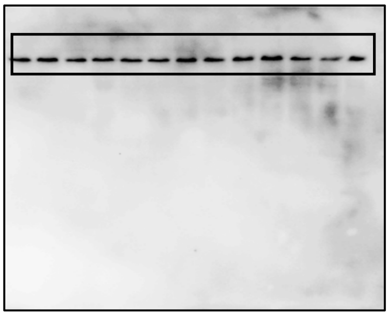

PCNA (bottom) α-tubulin(bottom)

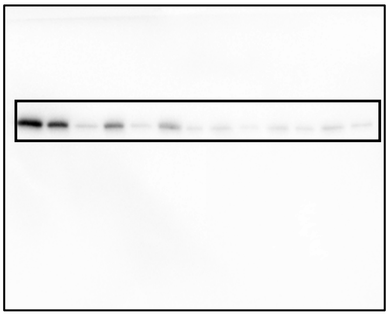

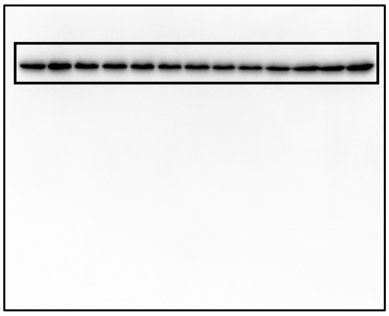

Supplementary Fig.4A

RB7LP α-tubulin

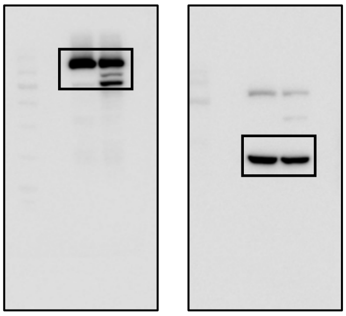

Supplementary Fig.5A

RB1 **(MIA PaCa-2, PK-45H，PK-1)**

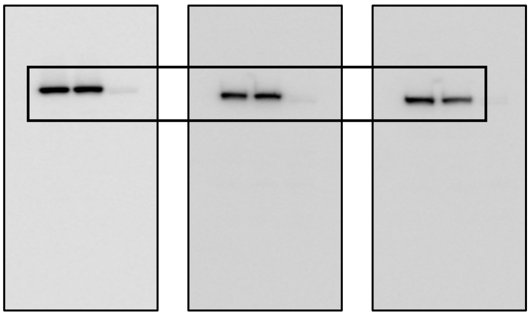

α-tubulin **(MIA PaCa-2, PK-45H，PK-1)**

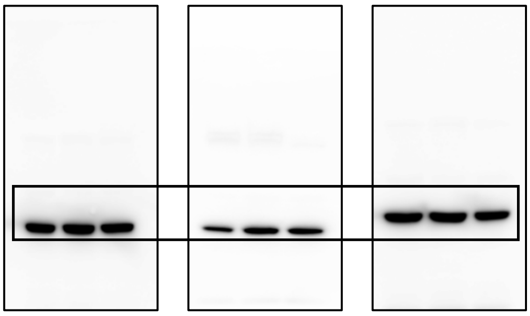

Supplementary Fig.6B

GTP-KRAS

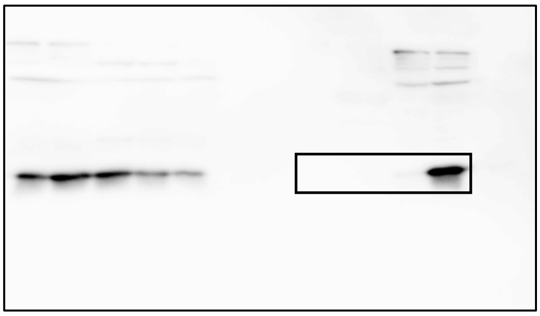

Supplementary Fig.7A

MIA PaCa-2(left)

p-EGFR EGFR p-ERK1/2

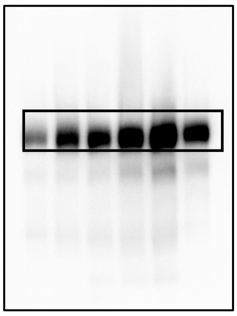
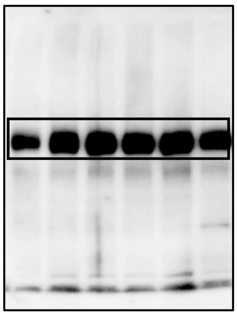
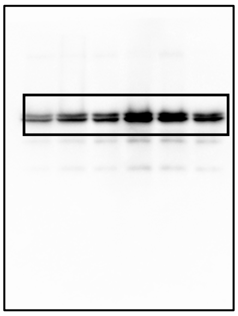

ERK1/2 HSP90

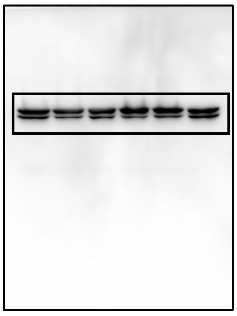
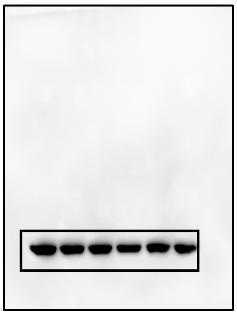

PK-45H(middle)

p-EGFR EGFR p-ERK1/2

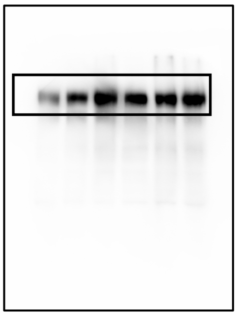
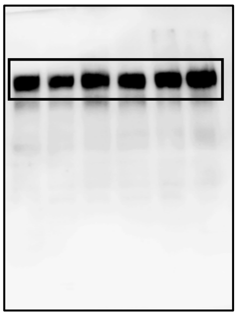
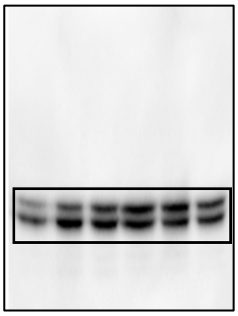

ERK HSP90

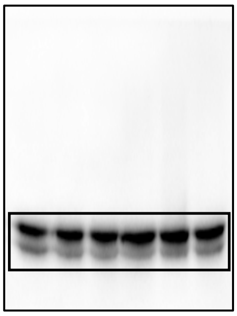
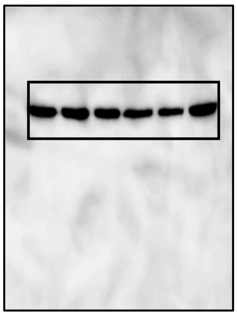

PK-1(right)

p-EGFR

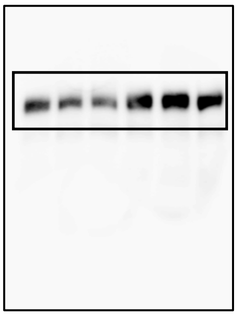

EGFR

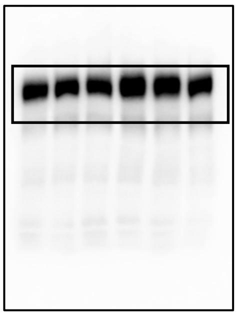

p-ERK1/2

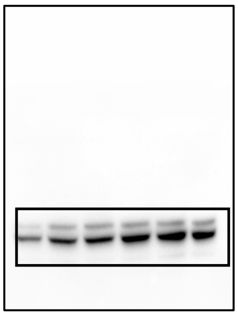

ERK1/2

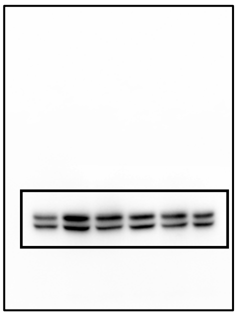

HSP90

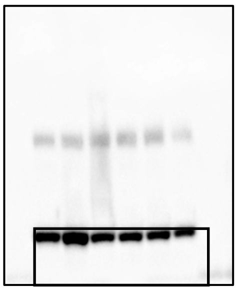

Supplementary Fig.7C

p-EGFR

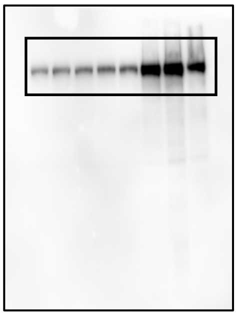

EGFR

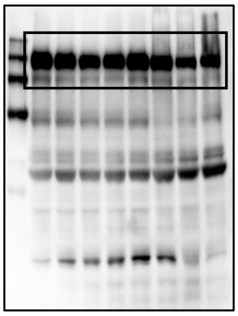

α-tubulin

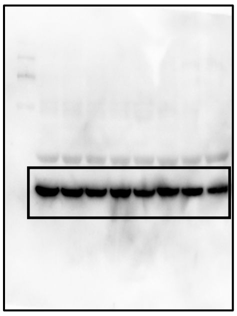

Supplementary Fig.8A

EGFR

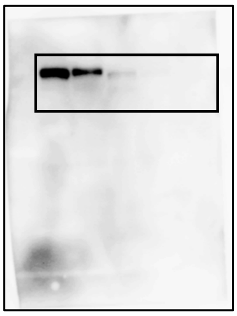

HSP90

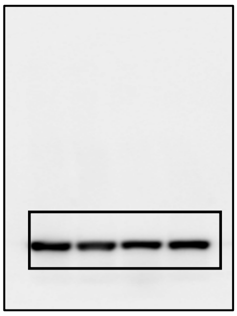

Supplementary Fig.8C

γH2A.X

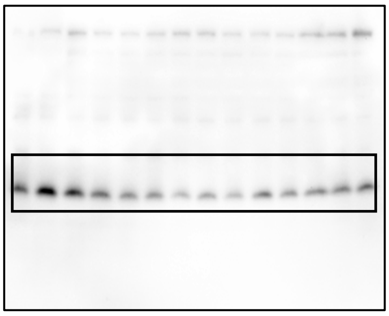

p21WAF1

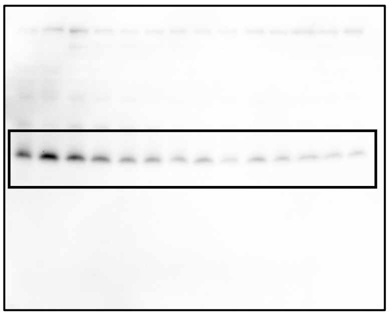

α-tubulin

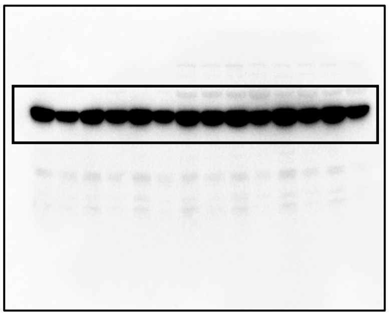

Supplementary Fig.9G

γH2A.X

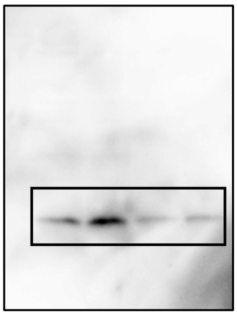

p21WAF1

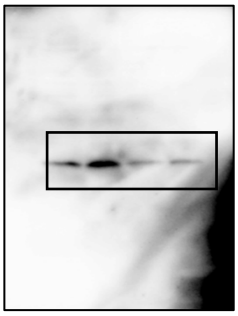

Bcl-2

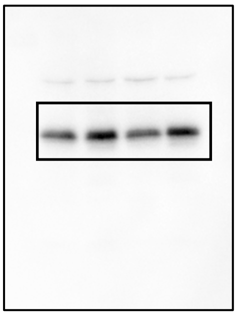

Bcl-xL

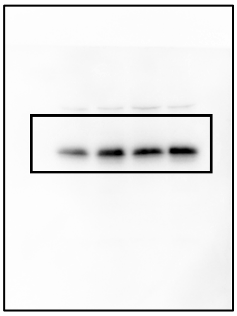

α-tubulin

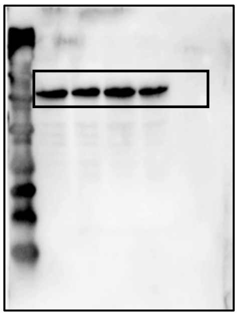

Supplementary Fig.10A

p-EGFR

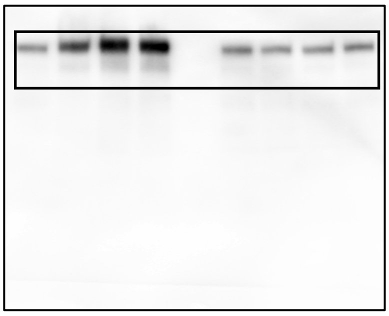

EGFR

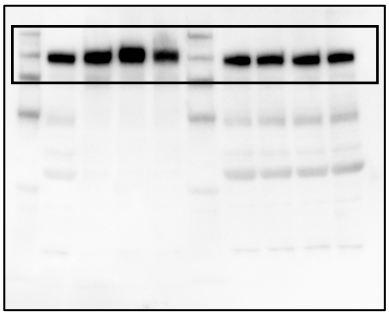

α-tubulin

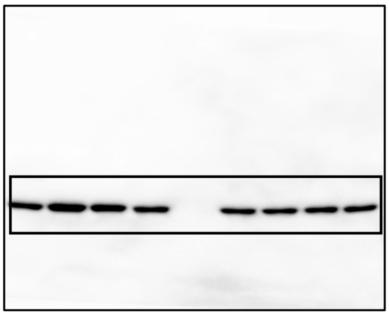

Supplementary Fig.11A

p-RB1

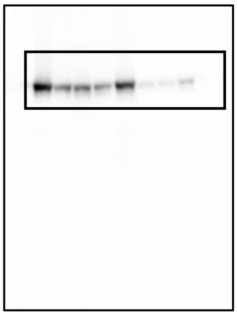

RB1

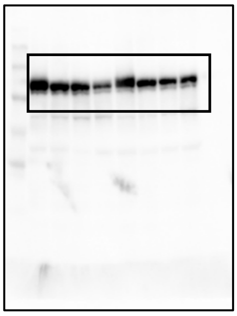

p-EGFR

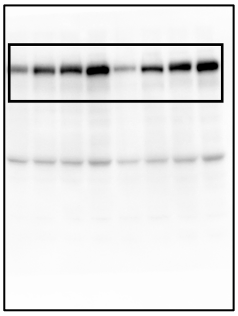

EGFR

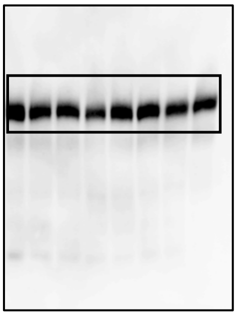

p-ERK1/2

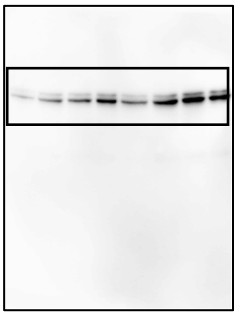

ERK1/2

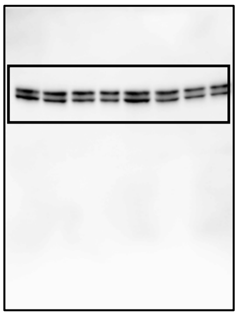

HSP90

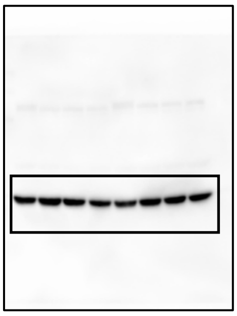

Supplementary Fig.12B

p-EGFR

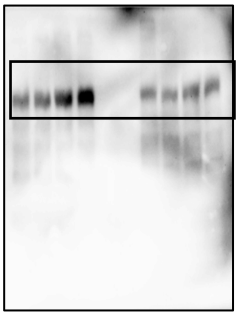

EGFR

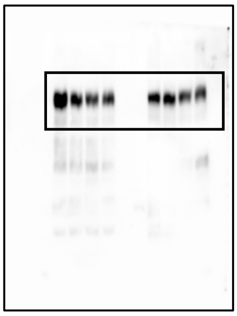

HSP90

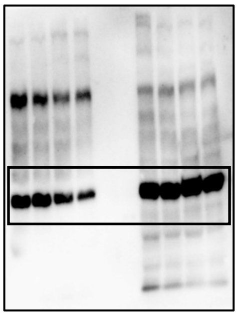

Supplementary Fig.12C

p-ERK1/2

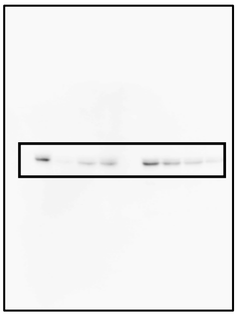

ERK1/2

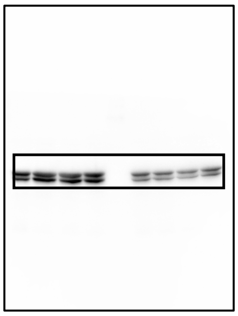

α-tubulin

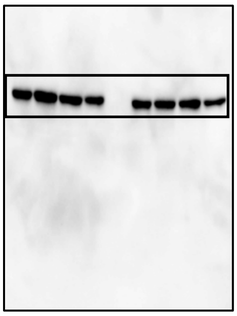

Supplementary Fig.13D

phospho-AMPKα

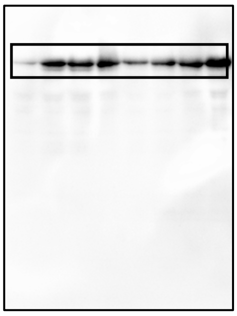

AMPKα

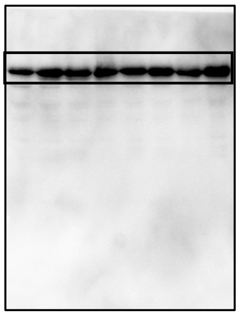

p-SAPK/JNK

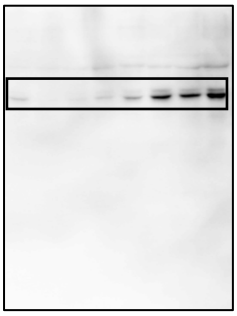

SAPK/JNK

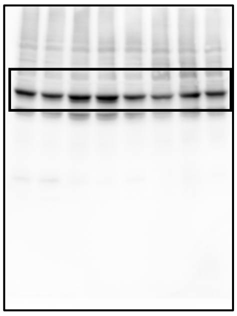

p-p38 MAPK

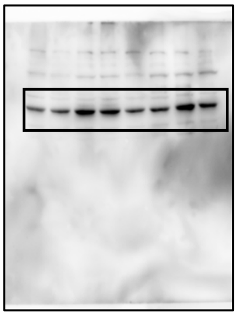

p38 MAPK

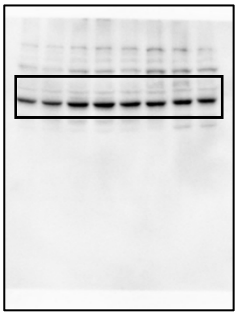

α-tubulin

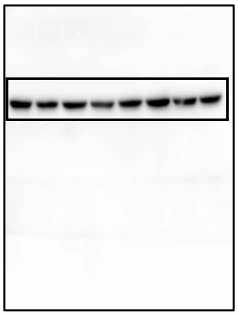

Supplementary Fig.13E

p-SAPK/JNK SAPK/JNK α-tubulin

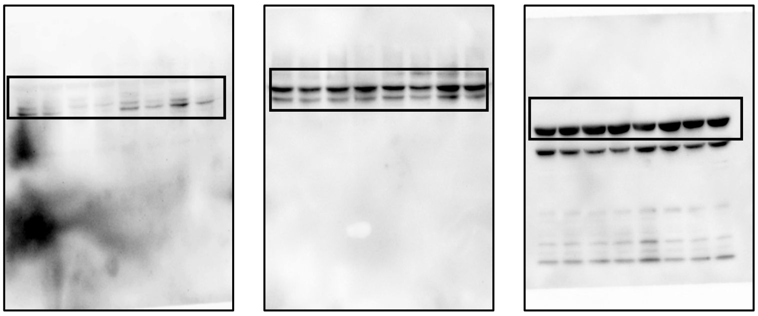

Supplement: Supplementary file 3 — Supplementary data UNCROPPED IMMUNOBLOTTING [file 41418_2025_1634_MOESM3_ESM.pdf]
