# Supplementary material for: Deprivation of EGFR signal causes senolysis in PDAC with CDK4/6 inhibition
Source: Cell Death Differ. 2025 Dec 18;33(6):1218–33. doi: 10.1038/s41418-025-01634-0 (PMC13246951; doi:10.1038/s41418-025-01634-0)
Supplement: Supplementary file 4 — Supplementary information ABBREVIATIONS [file 41418_2025_1634_MOESM4_ESM.pdf]

ABBREVIATIONS

Deprivation of EGFR signal causes senolysis in PDAC with CDK4/6 inhibition

Yuanyuan Zhang[1], Susumu Kohno[1], Keqi Gao[2], Mahadi Hasan[3], Tomohisa Baba[2], Zixue Zhang[1,2], Nao Sankoda[4], Hai Yu [1], Junjian Pan [1], Noriko Gotoh[5], Makoto Nakanishi[6], Yasuhiro Yamada[4], Jindan Sheng[1,7,8,9], Takiko Daikoku[3], Yoshikazu Johmura[2] and Chiaki Takahashi[1,*]

[1]Division of Oncology and Molecular Biology, Cancer Research Institute, Kanazawa University, Kanazawa, Ishikawa 920-1192, Japan. [2]Division of Cancer and Senescence Biology, Cancer Research Institute, Kanazawa University, Kanazawa, Ishikawa 920-1192, Japan. [3]Division of Animal Disease Model, Research Center for Experimental Modeling of Human Disease, Kanazawa University, Kanazawa, Ishikawa 920-8640, Japan.[4]Department of Molecular Pathology, Graduate School of Medicine, The University of Tokyo, Bunkyo-ku, Tokyo 113-0033, Japan[5]Division of Cancer Cell Biology, Cancer Research Institute, Kanazawa University, Kanazawa, Ishikawa 920-1192, Japan.[6]Division of Cancer Cell Biology, Institute of Medical Science, The University of Tokyo, Tokyo, Minato-ku, 108-8639, Japan.[7]Maternal-Fetal Medicine and Gynecologic Oncology, Shanghai First Maternity and Infant Hospital, School of Medicine, Tongji University, Shanghai, 200092, China.[8]Department of Gynecology, Shanghai First Maternity and

Infant Hospital, School of Medicine, Tongji University, Shanghai, 200092, China. [9]Dana-Farber Cancer Institute, Harvard Medical School, Boston, MA 02215, USA.

* Corresponding author:

Chiaki Takahashi, Cancer Research Institute, Kanazawa University, Japan; Tel: +81-76-264-6750; Fax: +81-76-234-4521; E-mail: chtakaha@staff.kanazawa-u.ac.jp

Abbreviations

The abbreviations for terms described in the manuscript.

AMPK	AMP-activated protein kinase
Bcl-2	B-cell lymphoma-2
Bcl-xL	B-cell lymphoma-XL
CDK	Cyclin-dependent kinase
CDKN2A	Cyclin-dependent kinase inhibitor 2A
CHK2	Checkpoint kinase 2
CDX	Cell line-derived xenograft
CK19	Cytokeratin 19
CM	Conditioned medium
DEGs	Differentially expressed genes
DOX	Doxycycline
EGFR	Epidermal growth factor receptor
ER	Estrogen receptor
ERK	Extracellular signal-regulated kinase
GSEA	Gene set enrichment analysis
GST	Glutathione-S-transferases
HER	Human epidermal growth factor receptor
HR	Hormone receptor
IB	Immunoblotting
MAPK	Mitogen-activated protein kinase
MitoQ	Mitoquinone
KRAS	Kirsten rat sarcoma viral oncogene homolog
NF-κB	Nuclear factor kappa B
NAPSA	Napsin A aspartic peptidase
NSCLC	Non-small cell lung cancer
OXPHOS	Oxidative phosphorylation
p21/WAF1/CDKN1A	Cyclin-dependent kinase inhibitor 1A
PanIN	Pancreatic intraepithelial neoplasia
PARP	Poly ADP-ribose polymerase
PCNA	Proliferating cell nuclear antigen
PDAC	Pancreatic ductal adenocarcinoma
PI	Propidium iodide
RB1	Retinoblastoma 1
RBD	RAS-binding domain
JNK	C-Jun N-terminal kinase
SASP	Senescence-associated secretory phenotype
SA-β-gal	Senescence-associated β-galactosidase
SCLC	Small cell lung cancer

Continued

SPRED1	Sprouty related EVH1 domain containing 1
TGF-α	Transforming gowth factor alpha
SPRY	Sprouty homolog
γH2A.X	γ-H2A histone family, member X
